# Laboratory exploration of mineral precipitates from Europa’s subsurface ocean

**DOI:** 10.1107/S1600576721008554

**Published:** 2021-09-29

**Authors:** Stephen P. Thompson, Hilary Kennedy, Benjamin M. Butler, Sarah J. Day, Emmal Safi, Aneurin Evans

**Affiliations:** a Diamond Light Source, Harwell Science and Innovation Campus, Didcot, Oxfordshire OX11 0DE, United Kingdom; b School of Ocean Sciences, Bangor University, Menai Bridge, Anglesey LL59 5AB, United Kingdom; c Environmental and Biochemical Sciences, The James Hutton Institute, Craigiebuckler, Aberdeen AB15 8QH, United Kingdom; d Astrophysics Group, Lennard-Jones Laboratories, Keele University, Keele, Staffordshire ST5 5BG, United Kingdom

**Keywords:** Europa, icy moons, low-temperature mineral precipitation, ocean worlds, long-duration studies

## Abstract

The precipitation of hydrated phases from a chondrite-like Na–Mg–Ca–SO_4_–Cl solution is studied using *in situ* synchrotron X-ray powder diffraction, under rapid- (360 K h^−1^, *T* = 250–80 K, *t* = 3 h) and ultra-slow-freezing (0.3 K day^−1^, *T* = 273–245 K, *t* = 242 days) conditions. The precipitation sequence under slow cooling initially follows the predictions of equilibrium thermodynamics models. However, after ∼50 days at 245 K, the formation of the highly hydrated sulfate phase Na_2_Mg(SO_4_)_2_·16H_2_O, a relatively recent discovery in the Na_2_Mg(SO_4_)_2_–H_2_O system, was observed. Rapid freezing, on the other hand, produced an assemblage of multiple phases which formed within a very short timescale (≤4 min, Δ*T* = 2 K) and, although remaining present throughout, varied in their relative proportions with decreasing temperature. Mirabilite and meridianiite were the major phases, with pentahydrite, epsomite, hydrohalite, gypsum, blödite, konyaite and loweite also observed. Na_2_Mg(SO_4_)_2_·16H_2_O was again found to be present and increased in proportion relative to other phases as the temperature decreased. The results are discussed in relation to possible implications for life on Europa and application to other icy ocean worlds.

## Abstract 2

Precipitation experiments from a model Europan ocean solution subjected to fast and slow freezing suggest that the highly hydrated Na–Mg sulfate phase Na_2_Mg(SO_4_)_2_·16H_2_O is one of the lowest-temperature mineral phases likely to be stable on Europa’s surface and may therefore be astrobiologically significant.

## 1. Introduction   

Jupiter is the largest planet in the Solar System and has 79 currently identified moons (https://solarsystem.nasa.gov/moons/jupiter-moons/overview). Europa is a lunar-sized body orbiting between the moons Io and Ganymede. Along with Calisto, these are the largest of Jupiter’s moons. The four are known collectively as the Galilean moons, after Galileo Galilei who, in 1610, was the first both to observe them and to recognize that they were satellites to another planet. However, despite these early observations, the existence of a global ocean of liquid water beneath an icy surface is a relatively recent discovery.

**Figure 1:**
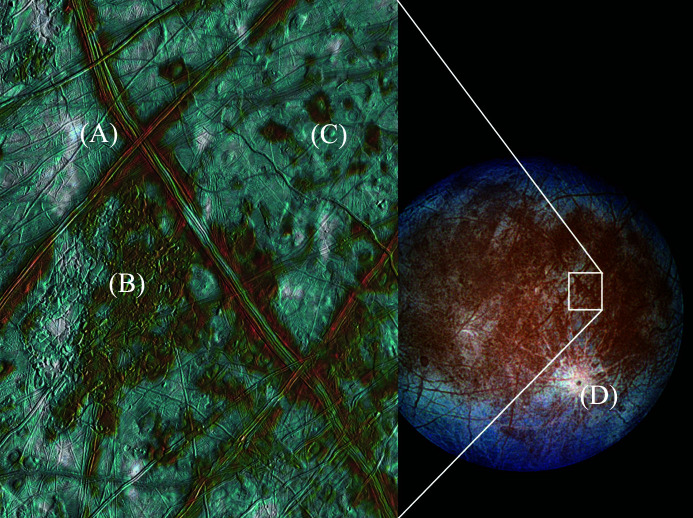
False-colour image of the surface of Europa taken by the Galileo spacecraft. Red–brown areas are non-ice material resulting from surface geological activity. Icy plains are shown in blue tones. Also visible are (A) long, dark parallel lines comprising ridges and fractures in the crust, (B) chaos terrain, (C) domes, and (D) impact craters. Courtesy of NASA/JPL/UArizona.

Images from the Voyager missions of the 1970s revealed a surface with relatively few craters, indicative of recurrent global resurfacing. Both Voyager and the later 1990s Galileo mission showed the surface of Europa to be smooth and criss-crossed by extensive intersecting fractures (Fig. 1 ▸), along with other surface structures with diverse morphologies (Pappalardo *et al.*, 1999 ▸). While the surface itself is completely frozen, its geology appears consistent with the presence of a subsurface liquid layer; for example, in places blocks of crust give the appearance of having been pulled apart and rotated within a slushy or liquid medium. Measurements of the density of Europa suggest a layered interior consisting of a rocky core (possibly with a differentiated inner core of iron), an H_2_O-rich layer and an ice crust (Nimmo & Manga, 2009 ▸; Schubert *et al.*, 2009 ▸).

As with the other Galilean moons, Europa probably formed ∼4.5 Ga from leftover material following the condensation of Jupiter from gas and dust in the early solar nebula, with models (*e.g.* Travis *et al.*, 2012 ▸) suggesting that ocean formation occurred within the first 0.5 Ga. However, the paucity of large (>10 km) impact craters coupled with a diverse range of surface morphologies points to a dynamic evolution of Europa’s ice shell in geologically recent times (∼60 Ma). These are the outward manifestations of both the internal structure and subsurface processes of the moon. Spectroscopic observations (Dalton *et al.*, 2005 ▸) suggest the presence of an extensive surface mineralogy composed of hydrated salts deriving, at least in part, from the delivery of oceanic water to the surface. As such, the surface mineralogy should provide clues to the subsurface environment and its processes. However, the data thus far do not provide for definitive identifications, owing to a combination of factors – the relatively low resolution of the Galileo instrument, the high noise content of the data themselves and the limited number of available library spectra used to make comparisons. Other investigative routes, such as laboratory experimentation, are therefore required in order to provide further physical insights and constraints, as well as to identify potential candidate phases for spectral fitting. For example, it was established early on that the rapid freezing and thermal cycling of dilute aqueous solutions of Na_2_SO_4_, MgSO_4_ and Na_2_CO_3_ produced materials that gave near-infrared reflectance spectra that, although distinct from crystalline minerals, bore a resemblance to the spectra of Europa’s surface material (McCord *et al.*, 2002 ▸).

### 1.1. The Europan ocean   

Evidence of a subsurface ocean was provided by magnetometer readings from the Galileo spacecraft during five close flybys (<2000 km) between 1996 and 2000. Deformations in the geometry of Jupiter’s magnetic field were detected that were consistent with the movement, within the field, of a conducting object (Khurana *et al.*, 2009 ▸). The perturbations could be fitted to the volume and conductivity of the conducting medium, showing that this could be neither at the centre of the moon nor deep within a rocky core, but rather within ∼30 km of the surface (Zimmer *et al.*, 2000 ▸). Furthermore, the electrical conductivity was suggestive of liquid water with a salinity close to that of terrestrial seawater. Although surface spectra from Galileo were noisy, owing to the intense radiation generated by the magnetic field of Jupiter, they did contain indications of salts consistent with the extrusion of liquid saline water (Dalton *et al.*, 2005 ▸). Early proposals for the composition of Europa’s ocean centred around three main possibilities, a neutral Na–Mg–SO_4_–H_2_ solution, an alkaline Na–SO_4_–CO_3_ solution or an acidic Na–H–Mg–SO_4_ system (Kargel *et al.*, 2000 ▸; Marion, 2001 ▸, 2002 ▸; Kempe & Kazmierczak, 2002 ▸).

The pressure at the base of the water layer is within the field of normal ice such that, unlike on a number of other icy moons (*e.g.* Ganymede, see Section 4.5.2), the liquid ocean is not sandwiched between low- and high-pressure ice phases, but rather should be in direct contact with the rocky core of the moon (Kuskov & Kronrod, 2005 ▸). The core itself is expected to be of chondritic silicate composition, being derived from pre-solar materials (Fanale *et al.*, 2001 ▸). Although long-lived radioactive elements are likely to be contained within the core, along with residual heat left over from Europa’s collisional formation from smaller bodies, calculations suggest that these are insufficient to maintain a liquid ocean of the volume deduced from the Galileo measurements (Schubert *et al.*, 2009 ▸). Instead, the main source of heating is the tidal extraction of energy from Europa’s eccentric orbit. Although this should, over time, circularize the orbit, the relationship between the orbital periods of Io, Europa and Ganymede (the orbital period of Europa is twice that of Io and half that of Ganymede) allows the eccentricity to be maintained (Sotin *et al.*, 2009 ▸) and provides an ongoing supply of energy capable of sustaining a liquid ocean.

The prospect of a global subsurface ocean that has remained liquid up to the present day makes Europa a prime target in the search for life beyond Earth (Marion *et al.*, 2003 ▸; Greenberg, 2008 ▸). As a potential abode for life, Europa meets the three key criteria for habitability: (i) a source of energy, (ii) liquid water and (iii) the availability of biologically essential elements (Priscu & Hand, 2012 ▸). Underlying this last criterion is the primary fact that the ocean maintains contact with the rocky core, whose chondritic composition means it will be rich in biologically essential elements. As proposed for Earth (*e.g.* Martin *et al.*, 2008 ▸), hydrothermal vents on the sea-floor could provide the original environment in which Europan life could potentially have developed, fuelled by chemosynthesis or serpentinization (Henin, 2018 ▸). These same chondritic materials are of course the source of the salinity of Europa’s ocean (see Section 1.3) and, in terms of habitability, the ocean itself is not a particularly extreme environment. Ocean temperatures should lie close to freezing, though the salinity will probably reduce these to around 250 K. Moreover, despite the ocean being ∼100 km deep, the sea-floor pressure should only be ∼110 MPa because Europa’s gravity is less than one-seventh of Earth’s. In this respect, it is equivalent to the pressure in the 11 km-deep Mariana Trench on Earth, which, though extreme by Earth standards, is known to support an active and diverse microbial ecology (*e.g.* Takami *et al.*, 1997 ▸; Nunoura *et al.*, 2015 ▸, 2018 ▸; Tarn *et al.*, 2016 ▸). The current observational pressure limit for life on Earth is ∼150 MPa (Hazael *et al.*, 2016 ▸), while experiments show that viable prokaryotic cells can survive pressurization (at least over laboratory timescales) into the 2–3 GPa range under static compression. Higher species such as liparid snailfish have also been observed within the Mariana Trench at depths of 8.1 km (Linley *et al.*, 2016 ▸), equivalent to a pressure of ∼82 MPa. Their likely maximum depth (∼8.4 km) is limited by biochemical factors concerning osmotic regulation in teleost taxa, related to their evolutionary origins in fresh water environments, rather than pressure per se (Yancey *et al.*, 2014 ▸) (note also that deep-sea fish lineages on Earth probably represent a recolonization from shallower depths following an anoxic extinction event during the Cretaceous period; Priede & Froese, 2013 ▸).

### 1.2. Ocean–surface transport   

Five predominant Europan surface terrains have been identified: (i) chaos terrain, (ii) ridges, (iii) plains, (iv) bands and (v) crater terrain (Figueredo & Greeley, 2000 ▸, 2004 ▸; Greeley *et al.*, 2000 ▸). Of these, chaos and ridged plains cover the majority of the surface (Doggett *et al.*, 2009 ▸; Greeley *et al.*, 2000 ▸; Schenk, 2009 ▸), which has led to numerous competing hypotheses to explain their origin and relation to the underlying ocean. For example, for chaos terrain these have included melt-through (Greenberg *et al.*, 1999 ▸; O’Brien *et al.*, 2002 ▸), diapirism (Pappalardo *et al.*, 1998 ▸; Schenk & Pappalardo, 2004 ▸) and the collapse of a melt-lens within the ice shell (Schmidt *et al.*, 2011 ▸; Walker & Schmidt, 2015 ▸); while for double ridges proposals include cryovolcanism (Fagents *et al.*, 1997 ▸; Kadel *et al.*, 1998 ▸), tidal squeezing (Greenberg *et al.*, 1998 ▸), linear diapirism (Head *et al.*, 1999 ▸), shear heating (Nimmo & Gaidos, 2002 ▸), compression (Sullivan *et al.*, 1998 ▸), wedging (Melosh & Turtle, 2004 ▸; Han & Melosh, 2010 ▸; Johnston & Montési, 2014 ▸) and compaction (Aydin, 2006 ▸); and for ridge-and-trough terrain, extensional tilt-blocks (Kattenhorn, 2002 ▸) and folding (Leonard *et al.*, 2015 ▸). As well as impact craters, lenticular structures (from the Latin *lenticulae* meaning ‘freckles’) are dotted across the surface of Europa and could be suggestive of localized lava domes due to eruptions of viscous icy slurries, successive stacking of thin fluid flows, inflation of ice-covered water flows, or surface breaching by warm ice diapirs and subsequent flow, or relaxation, of the ice for short distances over the surface.

These morphological interpretations place constraints on the ice-shell thickness, which is the limiting factor in whether the hypotheses provide for physical ocean-to-surface links to allow oceanic materials to be delivered to the surface. In general, only thin-shell models provide for direct ocean–surface contact. Models of Europa’s orbit imply that its eccentricity and obliquity periodically vary over geological timescales (Hussmann & Spohn, 2004 ▸; Bills *et al.*, 2009 ▸) and, since tidal heating and the resulting equilibrium shell thickness both depend on these two orbital elements, the thickness of the Europan ice shell is likely to vary over time. There should, therefore, be significant periods when the thickness lies somewhere between two thick/thin extremes. Unfortunately, the shell thickness is poorly constrained by both observations and models. Nevertheless, the expected tidal and radiogenic heat for Europa predict a thickness of 20–30 km (Hussmann *et al.*, 2002 ▸; Spohn & Schubert, 2003 ▸), while the physical properties of ice (rheology and grain size) suggest that convection within the shell should initiate for thicknesses of 15–25 km (McKinnon, 1999 ▸). The two largest multi-ringed craters on Europa similarly point to a thickness of ∼20 km at their time of formation (Schenk, 2002 ▸). More recent analysis of likely tidal heating, dissipation and conductive cooling suggests an average thickness of 15–35 km (Quick & Marsh, 2015 ▸; Vilella *et al.*, 2020 ▸). Furthermore, investigation of previously unanalysed high-resolution images from Galileo suggests that the local-scale resurfacing has evolved over time, transitioning from distributed deformation (expressed by the formation of the ridged plains) to discrete deformation (typified by the formation of chaos terrain and isolated fractures), and is probably consistent with progressive shell thickening and cooling (Leonard *et al.*, 2018 ▸).

**Figure 2:**
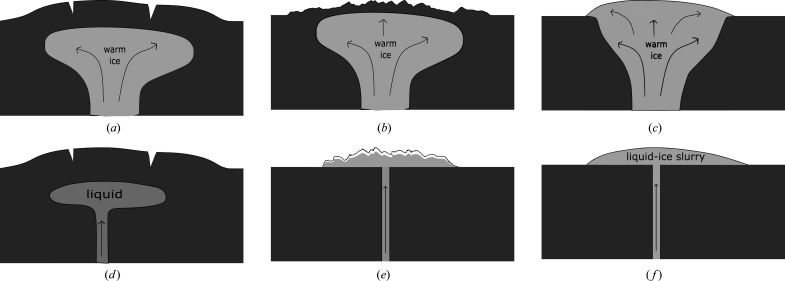
Origin of Europan surface morphologies I: formation of positive surface relief features on Europa involving potential delivery of subsurface ice–water to the planet’s surface [figures after Fagents (2003 ▸)]. Top panel: diapirism in a thick ice shell leading to (*a*) surface up-warping, (*b*) surface disruption, or (*c*) surface breaching and viscous flow. Surface up-warping and disruption by a rising diapir could also be accompanied by melting and release of near-surface brines (Head & Pappalardo, 1999 ▸). Bottom panel: intrusion of injected ocean water into a thin ice shell leading to (*d*) surface up-warping, (*e*) ice-covered effusions or (*f*) viscous extrusions causing cryolava domes.

In terms of more localized liquid water existing just below the surface, Schmidt *et al.* (2011 ▸) proposed that large-scale chaos features could be explained by near-surface liquid water (but without complete melt-through of the ice shell), while Manga & Michaut (2017 ▸) proposed a model of microfeature formation that implied present-day near-surface liquid water underneath all pits, some domes and some small chaos features. Similarly, ridge formation proposals include moving liquid water (Dombard *et al.*, 2013 ▸; Craft *et al.*, 2016 ▸), freezing within the shell (Johnston & Montési, 2014 ▸), and large-scale ice-plate motion and subduction (Kattenhorn & Prockter, 2014 ▸). For shell thicknesses > 20 km, whole surface breaching or melt-through by sporadic impacts is unlikely (Schenk, 2002 ▸) but could cause localized melts, or breaches of liquid-filled subsurface sills, exposing their contents to freezing surface conditions. Graphical representations of some of the many proposed ocean-to-surface mechanisms are given in Figs. 2 ▸–5 ▸ ▸ ▸.

The forgoing discussion is not intended as a complete or exhaustive review. However, it should convey both the wide variety of processes and the range of conditions under which oceanic material could be transported to the surface, from locations where temperatures of 250–273 K allow water to remain liquid, to highly freezing conditions where temperatures typically range from 110 K at the equator to 50 K at the poles, and encompassing regions and temperatures in between (*e.g.* within rising thermal plumes or liquid/freezing sills). Clearly, there is likely to be a similarly wide range in the timescales in which cooling and freezing occur, from long-lived slow-cooling near-equilibrium processes, likely to occur in long-lived subsurface sills, to rapid, kinetically dominated, flash freezing in eruptive events, typified, for example, by recent observations of active plumes similar to those seen on Enceladus (Roth, Saur *et al.*, 2014 ▸; Roth, Retherford *et al.*, 2014 ▸; Sparks *et al.*, 2016 ▸, 2017 ▸; Jia *et al.*, 2018 ▸).

### 1.3. Ocean composition   

The chemical evolution of a planetary ocean is directed primarily by the bulk composition and thermal history of the planetary object (Sohl *et al.*, 2010 ▸). The bulk composition concerns the ratios between rock, water ice, non-water volatiles and organic compounds, while the thermal history affects the freezing–thawing cycles, degree of ice melting, extent and duration of chemical interaction between rock and liquid water, degassing of the deep interior, and secondary precipitation of organic and inorganic phases. The wide possible variation in these factors leads to a diverse range of possible evolutionary pathways among the ocean-bearing bodies of the Solar System [see reviews by Hussmann *et al.* (2006 ▸), Nimmo & Pappalardo (2016 ▸), Lunine (2017 ▸) and Mann (2017 ▸) for known, probable and plausible ocean-bearing Solar System bodies]. Since Europa is subject to ongoing tidal heating, this long-lived heat source means that it is likely to experience extensive and prolonged water–rock interactions somewhat akin to hydrothermal systems on Earth, as previously mentioned. Not only do these provide the Europan ocean with its salinity, but they are also considered to increase the possibility for life since hydrothermal systems on Earth have long been considered potential genesis locations in origin of life theories (Corliss *et al.*, 1981 ▸; Baross & Hoffman, 1985 ▸; Holm, 1992 ▸).

The potential chemical composition of the Europan ocean can be constrained through possible weathering/leaching reactions and the assumed composition of its carbonaceous chondrite core (McKinnon & Zolensky, 2003 ▸). From observational data, Hand & Chyba (2007 ▸) suggested a salinity in the range 3–15 g kg^−1^. To identify the main likely elemental components, Fanale *et al.* (2001 ▸) subjected a sample of the Murchison CM meteorite (carbonaceous chondrite) to hot-water leaching to simulate low- to moderate-temperature hydrothermal processing. CM chondrites, although known to have suffered some mineralogical alteration while on their parent bodies, are believed to have retained their original cosmochemical composition (McSween, 1979 ▸; Wasson & Kallemeyn, 1988 ▸). The leachates were subjected to a series of sequential fractional crystallization steps, producing a series of ices and brines. Analysis of the brine compositions identified two relationships: (i) for cations Mg ≃ Na > (Ca, K, Fe) and (ii) for anions SO_4_ ≫ Cl. Numerical modelling by Zolotov & Shock (2001 ▸), based on a range of chondritic meteorite types, provided general agreement with the results of Fanale *et al.* Zolotov & Shock’s suggested model ocean composition had the following molal concentrations (moles kg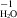):

(i) Cations: Mg^2+^ 6.271 × 10^−2^, Na^+^ 4.910 × 10^−2^, Ca^2+^ 9.637 × 10^−3^, K^+^ 1.964 × 10^−3^.

(ii) Anions: SO_4_^2−^ 8.744 × 10^−2^, Cl^−^ 2.087 × 10^−2^.

This corresponds to molal concentrations of soluble salts MgSO_4_ 6.271 × 10^−2^, NaCl 2.087 × 10^−2^, Na_2_SO_4_ 1.412 × 10^−2^, CaSO_4_ 9.637 × 10^−2^ and K_2_SO_4_ 9.8 × 10^−2^. The total salinity for this model is 12.3 g kg^−1^, which is almost three times less than terrestrial seawater. However, since chondrite meteorites will have suffered varying degrees of parent-body processing they may not fully represent the mineralogical make up of Europa’s primary material and could potentially produce unrepresentative leachates. McKinnon & Zolensky (2003 ▸), though, questioned the validity of an MgSO_4_-dominated ocean, including the assumed initial composition, the relevance of the leaching experiments and the difficulty of forming sulfate in reducing conditions. They suggested instead a ratio by weight of ∼10% water ice to ∼90% anhydrous rocky material with solar abundances of non-ice elements, which could yield an ocean with a lower sulfate concentration.

Although aqueous differentiation and long-term sea-floor leaching of Europa’s primary material is generally assumed to produce a system rich in sulfates, more extensive hydrothermal circulation and differentiation of the rocky component is more likely to result in an NaCl-rich ocean (Kargel *et al.*, 2000 ▸), as on Earth. The Saturnian satellite Enceladus, whose structure (rocky core + subsurface ocean + ice shell) may be similar to Europa’s, exhibits active plumes containing NaCl (but not Mg^2+^ or SO_4_^2−^; Postberg *et al.*, 2009 ▸, 2011 ▸), presumably arising from an NaCl-rich ocean (Waite *et al.*, 2006 ▸), and H_2_, suggestive of hydrothermal activity (Waite *et al.*, 2017 ▸). However, since the precise relationship between surface and ocean matter are not established, the presence of NaCl could represent either a body that has undergone more extensive processing than expected or simply the surface of a compositionally stratified ice shell (Zolotov & Shock, 2001 ▸). Furthermore, Na^+^ has been detected in Europa’s tenuous atmosphere (Brown & Hill, 1996 ▸), while Mg^2+^ has not (Hörst & Brown, 2013 ▸). Since the atmosphere results from the sputtering of surface material, this could either constrain the composition or support a compositional differentiation within the ice shell. However, chloride salts are spectrally inactive over the visible and much of the infrared, including the spectral regions utilized by many spacecraft and telescopes, which renders them both almost impossible to detect and incapable of accounting for the signatures of hydrated species thus far observed.

The above objections and observations concerning the possible limitations of leaching experiments notwithstanding, the likely chondritic nature of the initial building block materials of Europa makes a compelling case, and in the present work we assume (Section 2.1) an ocean composition closer to that predicted by leaching from a chondritic core.

### 1.4. Predicted precipitates from cooling Europan brines   

The behaviour of a cooling chondrite-based Europan ocean was modelled computationally by Kargel *et al.* (2000 ▸) using *FREZCHEM* (Marion *et al.*, 2010 ▸). This model assumed a Europan brine whose Mg–Na–Ca–sulfate composition (based on an earlier calculation; Kargel, 1991 ▸) was adjusted to include chlorides in the amounts determined by the Fanale *et al.* (2001 ▸) leaching experiments. For an initial solution of given composition, *FREZCHEM* minimizes the Gibbs free energy of the system. Solid phases formed during evaporation or freezing are assumed to have the potential to subsequently dissolve or react as the properties of the solution change, with all precipitated phases remaining available for subsequent reactions. This represents equilibrium crystallization and the recrystallizing phases can gain or lose waters of hydration, altering both the ionic composition and the concentration of the solution. Kargel *et al.* (2000 ▸) also investigated the possibility of fractional crystallization whereby precipitated phases are buried by other precipitating salts and therefore unavailable for any further reactions. In equilibrium freezing, magnesium sulfate first precipitates as MgSO_4_·7H_2_O, but this is completely replaced by MgSO_4_·11H_2_O via reaction with the brine as it cools, while in fractional crystallization MgSO_4_·7H_2_O is preserved and then buried by MgSO_4_·11H_2_O and other salts.^1^ The loss of sulfate enriches the remaining brine in chloride. The uptake of hydration waters and the precipitation of ice also contribute to this enrichment. The enrichment in Cl is particularly noticeable because, unlike all other ions, until hydrohalite precipitates at the eutectic, there is no removal of Cl from the brine.

Fractional and equilibrium crystallization produced very similar brine–precipitate sequences that varied only in the fine details of temperature and relative composition (plus an additional MgCl_2_·12H_2_O phase for fractional crystallization). The general order of precipitation with decreasing temperature for the Europan brine system predicted by Kargel *et al.* is




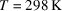




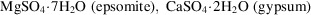




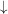




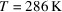




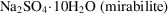




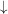




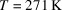




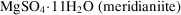




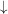




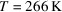




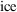




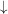




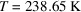




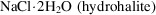




After hydrohalite the eutectic temperature is reached, below which liquid water cannot exist. The main feature of this sequence is that at higher temperatures the precipitates are dominated by sulfate phases. Both equilibrium and fractional crystallization showed an enrichment of chlorides at lower temperatures. The crossover between chloride and sulfate abundances occurs at about 266 K, close to the temperature where ice begins to precipitate, and following the point where the amount of water held in precipitated salts crosses over to exceed that held in liquid water (∼268 K). The colder the ultimate temperature, the higher the final Cl/SO_4_ ratio. These results were replicated by Marion *et al.* (2003 ▸, *e.g.* Fig. 5), while Zolotov & Shock (2001 ▸), using a more dilute starting solution, found MgCl_2_·12H_2_O was the last salt to precipitate, rather than NaCl·2H_2_O.

## 2. Experimental details   

Given the wide range of possible hydrated mineral phases within the Na–Mg–Cl–SO_4_–H_2_O system, the numerous complex pathways that exist between them and the varied means by which oceanic waters may be delivered to the surface, equilibrium thermodynamics may not be sufficient to model the behaviour of a large percentage of Europan oceanic waters once delivered to surface and near-surface regions. Consequently, in this paper the two experimental regimes of slow freezing and rapid freezing of a model Europan ocean solution (MEOS) are employed to probe the effects of equilibrium and non-equilibrium freezing conditions on precipitation behaviour.

### 2.1. MEOS composition   

As discussed in Section 1.3, Europa’s ocean probably lies within the Na–Mg–Ca–Cl–SO_4_–H_2_O system, though the precise relative concentrations are only weakly constrained. For the present work we have used a MEOS based on the general carbonaceous chondrite meteorite composition suggested by Kargel (1991 ▸) with a chloride content suggested by Fanale *et al.* (1998 ▸). The MEOS (1 kg) was prepared gravimetrically (to two decimal places, g) using distilled water, recrystallized analytical grade salts (Mg_2_SO_4_, Na_2_SO_4_ and NaCl) and an aqueous solution (∼1 mol kg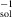) of CaCl_2_ (characterized by potentiometric titration; Papadimitriou *et al.*, 2013 ▸). The CaCl_2_ was prepared as a solution because of its hygroscopic nature in laboratory conditions. The MEOS was prepared to a molal composition (mol kg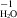) of Na = 1.630, Mg = 2.929, Ca = 0.0064, Cl = 0.308 and SO_4_ = 3.5964 (Marion *et al.*, 2005 ▸).

### 2.2. Data collection, reduction and analysis methodology   

*In situ* synchrotron X-ray powder diffraction measurements were performed on beamline I11 (Thompson *et al.*, 2009 ▸) at the Diamond Light Source. This is an undulator beamline with two experimental hutches in tandem. The first (EH1) houses a large three-circle diffractometer equipped with a fast position-sensitive detector (PSD) designed for *in situ* and *operando* experiments, particularly under non-ambient conditions (Thompson *et al.*, 2011 ▸). The PSD consists of 18 Mythen-II Si-strip detector modules tiled around a 90° arc. For the present work, to compensate for the small gaps between modules, while providing time and temperature resolution, each 10 s data set comprised two 5 s exposures, offset by 0.25° 2θ and automatically merged together by the data acquisition system. The X-ray energy was 15 keV (0.824603 Å, calibrated against NIST 640c Si reference powder). The second hutch (EH2) houses the Long-Duration Experiments (LDE) facility (Murray *et al.*, 2017 ▸) and is dedicated to the study of slowly evolving systems, again particularly under non-ambient conditions. Its operating energy is 25 keV (0.49388 Å, calibrated average for the experimental run), to provide cell/sample penetration, and detection is by area detector (Pixium), with 2D patterns obtained by integration around the image centre point. The LDE facility was used to house a cold cell designed for studies of hydrated mineral precipitation from slowly cooled aqueous solutions (Thompson *et al.*, 2018 ▸) (see also Section 2.2.2 below).

#### 2.2.1. Fast *in situ* cooling experiment   

Portions of the MEOS were injected into a 0.5 mm-diameter borosilicate capillary tube. This was placed within a brass holder and sealed at the holder end with wax. The other end was left open, with fluid being retained by capillary action, in order to avoid the build-up of excess pressure during isochoric freezing (*e.g.* Yakovlev & Downing, 2011 ▸). The holder was then placed on the spinner stage located at the centre of the EH1 diffractometer. An Oxford Cryosystems liquid nitrogen cryostream was mounted on a separate motorized table and aligned such that its nozzle enveloped the length of the capillary up to the point adjacent to where the X-ray beam impinges [Fig. 6 ▸ (top)]. The sample temperature was ramped from 256 K down to 80 K in 2 K steps (with 6 K min^−1^ ramp rate between steps, ±0.1 K at each step) and was allowed to equilibrate for 1 min at each step prior to data collection. The time–temperature profile for the experiment is shown in Fig. 7 ▸ (top).

*TOPAS* (Coelho 2018 ▸; http://www.bruker.com) was used to perform whole pattern Pawley fits to the diffraction data according to the following reductive procedure:

(i) Candidate phases with compositions based on the chemical composition of the solution were individually refined against the measured data.

(ii) Those that (*a*) provided fits to some of the Bragg peaks, (*b*) did not result in an unrealistically deviated background function in order to compensate for their intensity contribution and (*c*) did not introduce peaks in the fit that were absent in the data were accepted.

(iii) The phase with the lowest *R*_wp_ was taken as the starting fit and the remaining phases individually added in and refined against this.

(iv) Of these, the phase that then gave the best reduction in *R*_wp_ was retained and added into the fit.

This process was repeated until adding any of the remaining phases failed to improve the fit (*i.e.* increased *R*_wp_). The candidate phases and their starting lattice parameters were taken from published sources and the ICDD PDF4+ database (https://www.icdd.com/). The maximum 2θ was limited to ∼20° in order to prevent higher-angle data, where Bragg peaks from the various salt phases become severely overlapped, adversely influencing the fit.

#### 2.2.2. Slow *in situ* cooling experiment   

The LDE cold cell is shown in Fig. 6 ▸ (middle). The sample chamber [Fig. 6 ▸ (bottom)] is constructed from two bolt-together solid copper body components with two 6 mm-diameter circular apertures for beam transmission. The MEOS was contained by two 0.05 mm-thick diamond windows and two 0.5 mm-thick 7 mm-inner-diameter silicone O-rings, such that the sample fluids did not come into direct contact with the copper body [Fig. 6 ▸ (bottom) inset]. The sample blocks were encased by nine horizontally stacked Palight PVC foam boards, 25 mm thick, each having a divergent 0.025 mm Kapton window to provide an insulating multi-glazing effect while allowing diffracted X-rays to pass. A Lauda ECO RE1050 chiller unit (GOLD control head) was used to flow 60:40 glycol antifreeze and demineralized water refrigerant through the blocks in closed circuit. The cell temperature was thus regulated via the chiller, which was itself controlled externally, in open loop, using a computer script running on a dedicated EPICS input/output controller (https://epics.anl.gov/). Given a starting temperature, a ramp rate and the total number of days over which the cell is to be cooled, the script calculates and updates the required chiller setpoint temperature every 5 s to give a programmed cooling ramp. A rate of 0.3 K day^−1^ allows a reasonable difference in temperature to be achieved within the weekly measurement regime of the LDE facility [see Murray *et al.* (2017 ▸)]. Fig. 7 ▸(bottom) shows the time–temperature profile for the slow-cooling experiment.

Prior to the start of the experiment, the detector–sample distance was set to 350 mm, sufficient to give a reasonably wide 2θ range of 42° for energy calibration. The centre position of each cell was determined by scanning the cell chamber both horizontally and vertically through the beam and recorded in a data acquisition script. The chamber was then returned to the same position each week, and diffraction data were collected at this optimal in-beam position and at four additional vertical positions offset by ±1–2 mm. The beam size was 200 × 200 µm, achieved by slitting down the incident beam. Each week, using a built-in CeO_2_ reference standard, the wavelength and detector distances were automatically refined using a data reduction pipeline that incorporates the *DAWN* software suite (Filik *et al.*, 2017 ▸), which was also used to automatically integrate and convert the 2D images into conventional 2θ versus intensity ASCII files.

Large crystals forming in the sample chamber can result in very intense diffraction spots occupying a significant area of the detector. While it is, in principle, possible to mask these out prior to integration, the volume of data accumulated and the variability in their occurrence from week to week (such that a new mask would need to be drawn for each image) meant that this was not a feasible option in the first instance. Simple thresholding produced variable results such as halo artefacts, missed spots and loss of legitimate signals. Instead, as discussed by Thompson *et al.* (2018 ▸), the images were first integrated into separate 1D data files and visually inspected. Those showing broad and intense non-background features were rejected (typically 1–2 images per week), while the remaining data were averaged to produce a single data file for each week.

Data analysis for the slow-cooling experiment followed similar lines to that for the fast *in situ* experiment, but with several pre-processing steps. The wavelength, obtained from the CeO_2_ reference sample, was used in conjunction with the Bragg peaks from the diamond window of the cell to refine and fix the zero point for each weekly data set. Next, owing to the medium–low resolution nature of the data, only the low-angle peaks up to the first diamond peak at ∼14° 2θ were considered in the phase fitting process. This is because similarities in the symmetries and unit-cell dimensions of various salt phases yield highly overlapped peaks at higher angles, which do not allow for unique fits but can exert unwarranted statistical influence over the fit. At lower angles the Bragg peaks are sufficiently separated that qualitative phase identifications can be made, even if more quantitative fractional contributions to the scattering cannot be obtained from the fits.

## 3. Results   

For ease of presentation, we adopt the following abbreviations: sulfate and chloride phases will be denoted by *X*S*n* and *X*C*n* respectively, where *X* represents cations (M = Mg, N = Na and C = Ca) and *n* signifies the waters of hydration. For example, MgSO_4_·7H_2_O and NaCl·2H_2_O will be denoted by MS7 and NC2 *etc*. (Table 1 ▸).

### 3.1. Fast-freeze experiment   

Fig. 8 ▸ (top panel) shows three successive diffraction patterns separated in temperature by 2 K and in time by 4 min. Contrary to the predictions of equilibrium cooling (Section 1.4) the first crystalline phase observed here is hexagonal ice. However, these initial peaks are quickly followed by a complex collection of diffraction features from other solid phases. A waterfall-style plot of the diffracted intensity throughout the whole experiment [Fig. 8 ▸ (bottom)] shows that, once formed, all phases remain present throughout the experiment and no further phases are subsequently precipitated.

The scattering phases were identified [*e.g.* Fig. 9 ▸ (top)] using the fitting procedure outlined in Section 2.2.1 and are listed in Table 1 ▸. The temperature dependency of the scattered intensity for each phase is shown in Figs. 10 ▸ and 11 ▸, and Fig. 12 ▸ shows the corresponding percentage proportion that each phase contributes to the total (crystalline) scattered intensity. The intensity of a given phase depends on both the structure factor and the amount of the phase present, so while the relative proportions provide a simple approximation to the major/minor contributors to the scattering, in the absence of Rietveld fitting, relative qualitative changes in the individual phase abundances are better represented by changes in the intensities. The intensity plots have been grouped as follows:

(*a*) the two main contributors MS11 and NS10;

(*b*) NMS16 and NMS15, which show increasing intensity with decreasing temperature;

(*c*) MS7, NMS4 and CS2, which show no major change in intensity, along with MS5, which appears approximately constant below ∼130 K;

(*d*) NC2 and NMS5, which, along with ice, decrease in intensity with decreasing temperature.

By the end of the experiment the two main phases contributing to the diffracted signal are MS11 and NS10, which account for ∼45% of the scattered intensity. Two significant temperatures are apparent in the figures. Firstly, at about 200–220 K, many of the precipitated phases show either a maximum or a minimum in their intensities. Secondly, between 140 and 160 K, the intensities for several phases (*e.g.* MS11, NS10, NMS16 and NMS5) tend to either level off or decrease/increase more smoothly. Above ∼160 K, the relative collective behaviour of the precipitated phases appears complex, possibly suggestive of phase instabilities with significant solid–solid and solid–liquid interactions. In general, though, the overall trend throughout the experiment is for the minor phases with high hydration states (NMS15 and NMS16) to increase in proportion, while those of low or intermediate hydration (NMS5, NC2, CS2, MS7 and MS5) either decrease or remain essentially constant.

The two major phases MS11 and NS10, however, show a slightly different pattern of behaviour. NS10 initially shows a significant decrease until ∼200 K, below which its intensity recovers to a level higher than that of its initial formation, appearing to approach a steady state below 160 K. MS11 on the other hand is not a major component of the very first precipitate, but very quickly grows in intensity in the subsequent temperature step. However, as the temperature subsequently decreases it shows a slight oscillatory decline-and-recover behaviour, suggesting that at higher temperatures it may be unstable relative to other phases, until ∼180 K, where its intensity appears to level off. As the temperature drops below ∼160 K, MS11 shows a smooth recovery to just above its original high level.

The observation of NMS16 as a precipitate in this system is noteworthy as it is a hydrated phase that has only relatively recently been reported [observed to form from NMS4 at low temperature (Leftwich *et al.*, 2013 ▸); see the discussion in Section 4.1 below] and as such would not have been predicted by the *FREZCHEM* models, discussed in Section 1.4, that predated its discovery. The presence of this phase is shown by the appearance of a Bragg peak at ∼3.3° 2θ (*d* spacing = 14.252 Å) in Fig. 9 ▸ (top) and more clearly in Fig. 13 ▸. As a highly hydrated phase, NMS16 is also one of the phases whose intensity, and therefore quantity, appears to increase with decreasing temperature.

Zolotov & Shock (2001 ▸) predicted the formation of MgCl_2_·12H_2_O at the eutectic temperature of 237.05 K. However, as discussed above, following the initial freeze we do not observe the formation of any new phases. Moreover, even though the rapid-cooling experiment passed through the eutectic point 14 min after the initial 250 K starting temperature (where the sample was still liquid; see Section 4.1) and a base temperature of 80 K was reached 3 h 13 min later, we did not observe the formation of any MgCl_2_·*n*H_2_O (*n* = 2, 4, 6, 8, 12) phases. We do, however, observe the presence of NC2, which is consistent with the prediction (Section 1.4) of Marion *et al.* (2003 ▸).

### 3.2. Slow-freeze experiment   

Using the reduction and analysis procedure outlined in Section 2.2.2 [see also Fig. 9 ▸ (middle and bottom) for example fits], the following precipitation sequence was observed for the slow-freeze cold-cell experiment:
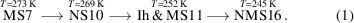
These are also indicated on the experiment’s time–temperature profile in Fig. 7 ▸. The order of precipitation up to MS11 follows that of equilibrium cooling (Section 1.4). This also predicts that ice is not the first solid phase to form, which, unlike in the rapid-freeze experiment, is what we observe. On the other hand we did not detect CS2 precipitation, but this could also be due to a combination of low Ca^2+^ concentration, formation of an amorphous precursor phase (see below) and/or small beam sampling (see discussion in Section 4.1). At ∼245 K the base temperature of the cold cell is above the predicted eutectic point for this system and consequently we do not observe NC2. However, after a long period of time at base temperature we do observe the formation of the highly hydrated NMS16 phase. Again, this is shown most clearly by the formation of a low-angle Bragg peak at ∼2° 2θ (*d* spacing = 14.19 Å) in Fig. 9 ▸ (bottom), which was absent from data collected earlier in the experiment [Fig. 9 ▸ (middle)].

It was not possible to extract detailed information on the relative time-varying abundance of each phase, as per the rapid-freeze experiment, owing to a combination of systematic and physical factors. Firstly, the formation of large crystals results in multiple large diffraction spots in the area detector images with diameters far wider than the powder rings, which varied in both size and position from week to week. As discussed, these images were rejected from the data reduction process. Secondly, the beam size necessary to obtain reasonable peak resolution in the LDE geometry was very small (200 × 200 µm) relative to the total sample volume (10 mm diameter × 1 mm thick). Therefore crystallites forming within the sample fluid could potentially sink out of view, resulting in sedimentation, particularly as ice is not the first phase to form. We assume that once ice formation takes place it occurs throughout the whole sample volume, such that any remaining high-salinity fluids are contained within inclusions within the ice. Precipitation within these could potentially lead to similar sedimentation effects. Although we have not measured the size of inclusions within our sample chamber, crystallite movement may be a reasonable assumption. Early studies on sea ice found brine pockets as small as a few times 0.1 mm in size (Perovich & Gow, 1996 ▸), while more recent magnetic resonance imaging of sea ice cores found brine inclusion diameters as large as ∼2–6.5 mm (Galley *et al.*, 2015 ▸). We assume therefore that brine pockets larger than the beam size could also exist within the cold cell following ice formation. Although these effects were mitigated by collecting data at multiple positions within each sample area, the automated nature of the LDE data collection process meant that these were (*a*) fixed positions and (*b*) limited in number (four locations for each of the five sample chambers of the cold cell, each running a separate experiment) owing to the constraints imposed by the time slot allocated to individual experiments running on the multi-experiment LDE facility (Murray *et al.*, 2017 ▸). The rejection of whole images ultimately results in a reduction in the gathered information, such that in some weeks a given phase, though present within the cell, could be under or over represented in the remaining data, or indeed in some instances not represented at all. Similarly, weak intermediate or metastable phases could be missed. However, since the precipitation order primarily follows the predicted equilibrium sequence, the likelihood of such phases forming may be small.

It is also conceivable that at these relatively high temperatures the cooling precipitate–brine system is relatively dynamic and that over the course of a week (the time between measurements, with Δ*T* = 2 K) a significant amount of reaction and/or exchange could occur between precipitated phases, and between the precipitates and the remaining brine (*e.g.* dissolution, loss or gain of H_2_O). In studies relating to surface salts on Mars, dehydration experiments at near- and sub-ambient temperature conditions (∼263–252 K) identified the formation of amorphous hydrated Mg sulfate (*e.g.* Vaniman *et al.*, 2004 ▸; Wang *et al.*, 2006 ▸; Dalton, 2012 ▸) and related phases (*e.g.* Sklute *et al.*, 2018 ▸). In the case of aqueous systems the nucleation of crystallites is a complex process that involves ion dehydration, close approach of like charges, and the arrangement of ions and molecules into ordered 3D structures (Kashchiev & van Rosmalen, 2003 ▸). The precipitation of amorphous phases on the other hand requires less stringent constraints (Vekilov, 2010 ▸) and therefore has long been considered a plausible precipitation route for many crystals, as has been extensively demonstrated for calcium carbonate and calcium phosphate (*e.g.* Addadi *et al.*, 2003 ▸; Günther *et al.*, 2005 ▸; Combes & Rey, 2010 ▸; Bots *et al.*, 1012 ▸). It is possible, therefore, that phases in the MEOS system could also precipitate via initially amorphous structures. Indeed, CS2 has been shown to precipitate via an amorphous CS phase, transforming to CS0.5 and subsequently CS2 (Wang *et al.*, 2012 ▸; Stawski *et al.*, 2020 ▸). Any putative amorphous phase that formed either by precipitation or by partial dissolution would be difficult to distinguish using X-rays but could react with both the remaining liquid brine and crystalline precipitates.

Qualitatively, the contribution of MS7 to the scattered intensity did appear, in general, to be reduced once MS11 had formed, consistent with the prediction that MS7 is replaced by MS11 (Kargel *et al.*, 2000 ▸). In our previous study of an MgSO_4_–H_2_O system using this sample cell (Thompson *et al.*, 2018 ▸), we similarly observed a slowly changing MS7/MS11 ratio and found that fits could be improved by the inclusion of an amorphous sulfate component to better model the background scatter. In addition, the strength of the background signal showed a steep rise below 250 K, which from the pattern of the diffuse scatter in the original area detector images was attributed to amorphous ice. This probably formed by residual water, with reduced salinity resulting from the precipitation of the salt, undergoing rapid freezing [see Fig. 8 of Thompson *et al.* (2018 ▸) and the discussion in Sections 4.3 and 5.1 of that paper]. Fig. 14 ▸ (top) shows the background signal for the slow-cooling MEOS experiment and records a similar steep rise in the region of 250 K, suggesting that a similar effect is in operation in this system.

The background signal for the rapidly cooled MEOS, however, shows a very different behaviour [Fig. 14 ▸ (top)]. Initially, in the liquid state, a high signal level is recorded, but this drops significantly over the course of three data collections as ice forms and salts subsequently precipitate, all within the space of a 4 K drop in temperature. Below ∼195 K [Fig. 14 ▸ (top, inset)], the background shows a smooth, but weak, rise as the temperature decreases further. Although the slow rise could be due to the release (and freezing) of water molecules as the various hydrated phases grow, shrink and transform *etc*., we suggest that the smooth variation is more characteristic of a solid-state structural effect caused by the freezing-in of static disorder, due to increases in molecular rigidity involving the waters of hydration, as the temperature drops. Studies of the ∼3500 cm^−1^ hydration band in the Raman spectra of various sulfates at room temperature report a band that is broad and smooth (*e.g.* Wang *et al.*, 2006 ▸). This is due to the stretching of O—H bonds, located within a range of crystallographically distinct sites, such that the shape of each bound water mol­ecule is severely distorted from that of a free water molecule. High numbers of structurally incorporated water molecules, coupled to a wide variation of O—H bond lengths, results in the smearing out of individual band components (Wang *et al.*, 2006 ▸). At lower temperatures, however, fine structure is observed to form within the band. A likely cause is a restriction in the ranges over which individual water molecules are distorted due to the relative strengthening of the hydrogen bonds as the temperature is decreased, which essentially leads to the water molecules becoming increasingly rigid. Under fast cooling, the rapid imposition of rigidity upon the water molecules should therefore introduce static disorder, giving rise to an increase in diffuse scatter.

## 4. Discussion   

### 4.1. Formation of Na_2_Mg(SO_4_)_2_·16H_2_O as a Europan mineral   

NMS16 was first reported by Leftwich *et al.* (2013 ▸) as a low-temperature phase derived by chance from deliquesced NMS4 held below 283 K for up to ten days in a freezer, its structure being solved using single-crystal X-ray diffraction. Subsequent attempts to form NMS16 resulted, 20–30% of the time, in a mixture of the single-cation phases NS10 and either MS11 or MS7, depending on the relative humidity and temperature conditions, suggesting that NMS16 was not thermodynamically stable (though the authors also considered the possibility that their NMS4 precursor could have decomposed into the single-cation phases prior to use). However, we observe the presence of NMS16 in both our fast- and our slow-freeze experiments. Following fast freezing, the phase initially appears to decrease in proportion down to ∼230 K (Fig. 12 ▸), suggesting that it may be unstable relative to the other phases at higher temperature but more thermodynamically favoured at low temperatures. Indeed, its intensity and relative proportion increase with falling temperature down to ∼160 K, where they level off until ∼110 K, below which they steadily increase as the temperature falls. This points to NMS16 being a stable low-temperature phase, but with slow nucleation and growth kinetics, perhaps limited by the stability (or meta­stability) of any precursor phase(s). In this respect NMS4 does not seem to be the necessary precursor, as below 160 K its intensity and relative proportion are constant (Figs. 11 ▸ and 12 ▸). Further evidence can be seen in Fig. 7 ▸ (bottom): for the slow-freeze experiment, NMS16 is not observed until ∼30 days or so after a base temperature of 245 K has been reached and long after the Mg-bearing MS7 has disappeared.

Low-temperature mineral transformations are generally thought to occur primarily by dissolution and precipitation reactions (*e.g.* Cole & Chakraborty, 2001 ▸; Putnis, 2009 ▸). These reactions can involve a mineral transforming from one phase to another, a mineral maintaining its structure and morphology but altering its elemental composition, or a mineral maintaining its structure and elemental composition but reforming into more stable particles through aggregation and growth (*e.g.* Ostwald ripening). Collectively, these reactions can lead to element- or nuclide-specific repartitioning between the aqueous and solid phases. While there are many different mineral growth, replacement and transformation mechanisms that can cause these changes, they are generally thought to occur when a system is at disequilibrium (Putnis, 2009 ▸; Yardley, 2009 ▸; Putnis & John, 2010 ▸; Putnis & Ruiz-Agudo, 2013 ▸).

In our experiments, NMS16 was clearly distinguished by the presence of a relatively weak Bragg peak at low angle (3.3° 2θ) in the fast-freeze experiment (see Fig. 13 ▸) and a much stronger peak, relatively speaking, at ∼2° 2θ in the slow-freeze experiment (this is the 001 reflection and the difference in peak positions is due to the difference in X-ray energy; the *d* spacings are 14.252 and 14.19 Å, respectively, consistent with differences in temperature, resolution and measurement geometry for the two experiments). Given what looks like a linearly increasing trend below 160 K for its intensity in the fast-freeze data (Fig. 10 ▸), it may be reasonable to speculate that, if the sample had been maintained at the base temperature beyond the existing end of the fast-freeze experiment, NMS16 would have become more abundant. Leftwich *et al.* (2013 ▸) conducted powder diffractometry experiments using powdered samples mounted on top a circulating methanol temperature-controlled stage inside a humidity-controlled cell, reporting that NMS16 persisted at temperatures as low as 150 K. Whether this was the limit of their apparatus or of the experimental programme itself is not clear, but the result is consistent with our own for aqueous precipitation. They also reported that transformation from their NMS4 precursor occurred between 243 and 263 K. This is consistent with the formation temperature of 245 K observed in the slow-freeze experiment, albeit with greatly differing timescales – Leftwich *et al.* observed NMS16 forming within minutes. NMS4 is only a minor component of the precipitate formed during the fast-freeze experiment (Fig. 12 ▸), while its presence was not detected in the slow-cooling experiment, which may indicate the presence of an alternative formation pathway.

Using Raman microspectroscopy, Vu *et al.* (2016 ▸) investigated Na–Cl–Mg–SO_4_–H_2_O brines frozen at 0.5 K min^−1^ with varying Na^+^:Mg^2+^ ratios of 2.13:1, 1:1 and 0.61:1, representing saturated, equimolar and oxidized–salty solutions, respectively. On the basis of the general positions of Raman features in certain characteristic spectral regions, they concluded that the precipitated phases were most likely hydrated sodium sulfate and chloride phases. Though they were unable to make definitive identifications, they attributed the sulfate to NS10 and the chloride to MgCl_2_ and NaCl. They also reported that these phases persisted down to 100 K. Repeating the measurements using compositions reported by Marion *et al.* (2005 ▸), Zolotov & Kargel (2009 ▸) and Zolotov (2012 ▸) yielded a similar conclusion, even for Na^+^:Mg^2+^ of 1:∼2 (*i.e.* similar to our MEOS). Noting the unexpected nature of this result and that forming a sodium sulfate requires two cations compared with magnesium sulfate’s one, to explain their results they hypothesized that magnesium sulfates have a significantly higher solubility at low temperature than sodium sulfates. This should make the sodium sulfate phases more likely to precipitate. Precipitation may also be aided by excess SO_4_^2−^ ions producing a common ion effect (Pape, 1981 ▸), which could also reduce the solubility of sodium sulfate. Although comparative magnesium and sodium sulfate solubility data at low temperatures are not available, investigations of terrestrial seawater brines have shown that sodium sulfate solubility changes significantly as a function of temperature between 273 and 252 K (Butler *et al.*, 2016 ▸).

Following on from their results, Vu and colleagues developed a thermodynamic chemical divide model (Johnson *et al.*, 2019 ▸) to predict Europan precipitates for an ocean with pH < 8.4 and their precipitation order, where the phases formed at each successive step are determined by a combination of their solubility and ionic compositions. However, more recent experimental work by the same authors (Vu *et al.*, 2020 ▸), combining Raman and X-ray diffraction, showed that the precipitated phases depend on both freezing rate and composition. They also found that multiple hydrated salts not predicted by the chemical models were frequently encountered in the final solid phase, and flash freezing of diluted brines often produced water ice together with amorphous hydrated Mg salts. Thus, while low temperatures may favour Na sulfate precipitation, the kinetics related to slower rates of temperature decrease may mean that Mg sulfate precipitation also becomes favourable. As such, the presence of NMS16 may be more reflective of a slower rate of cooling in both our experiments, rather than overall composition.

The observation of NMS16 by Leftwich *et al.* (2013 ▸) was made under ‘Mars relevant’ conditions, making it a candidate phase for detection on that planet. The observation here of NMS16 under Europa relevant conditions suggests that NMS16 may in fact be a ubiquitous feature throughout all cold Mg–Na saline environments.

### 4.2. Mineral stratification under slow freezing   

The velocity at which a salt particle precipitated out of solution will subsequently sink is described by Stokes law:
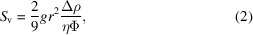
where 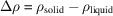 is the excess density of the solid particle relative to the solution, *g* the gravitational acceleration, η the dynamic viscosity of the solution, Φ the particle form (shape) resistance (*e.g.* oblate spheroid, rod, disc *etc*.) and *r* the radius of a sphere of equal volume [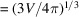, where *V* is the specific volume of the particle]. Assuming spherical particles, where *r* is the simple radius and Φ = 1, for a given viscosity, those particles with the highest excess density will thus achieve the highest sinking velocity. The densities of the various phases identified in this study are given in Table 1 ▸.

For a fixed-viscosity medium, assuming no potential mixing mechanisms such as liquid turbulence or convective motion, over time the particle depth distribution will become such that lower-density particles occupy lesser depths than higher-density ones. However, for water slowly approaching its freezing point η increases nonlinearly, going, for example, from 1.571 × 10^−3^ N s m^−2^ at 277 K to 1.792 × 10^−3^ N s m^−2^ at 273 K at normal pressure (https://www.engineeringtoolbox.com/water-dynamic-kinematic-viscosity-d_596.html), such that the sinking effect may be predominant only in slowly cooling solutions. Salinity increases the viscosity slightly [*e.g.* 1.67 × 10^−3^ N s m^−2^ at 277 K and 1.89 × 10^−3^ N s m^−2^ at 273 K for terrestrial seawater (https://www.engineeringtoolbox.com/sea-water-properties-d_840.html) with 35 g kg^−1^ salinity] while also depressing the freezing-point temperature. We assume, for the sake of argument, that the effect of MEOS salinity is similar and we ignore the viscosity-reducing effect that increases in pressure would have. In a solution that is freezing by reduction in temperature and therefore becoming increasingly viscous, two basic effects will come into play. Firstly, those phases that precipitate early on at higher temperature will do so in a less viscous medium and therefore sink farthest, in proportion to their densities, before being slowed by increased viscosity at lower temperature. Secondly, depending on the rate of cooling, the effect of excess density could subsequently deplete the locale of the heaviest phases should they precipitate early on. In our slow-freeze long-duration experiment, the temperature-dependent order of precipitation [equation (1) and Fig. 7 ▸] is such that MS7 (ρ = 1.679 g cm^−3^) and NS10 (ρ = 1.465 g cm^−1^) precipitate prior to ice, so that under the right cooling conditions MS7 could, relative to NS10, be preferentially lost to the solution that will subsequently become entrapped within the ice. The combination of cooling rate and measurement frequency of the slow-freeze experiment means that from the current data we cannot resolve the question of whether MS11 (ρ = 1.512 g cm^−3^) precipitates prior to, simultaneously with or after ice formation (as perhaps indicated by the fast-freeze data; Fig. 8 ▸). If MS11 forms just prior to ice, it too could be preferentially lost by sinking. The Europan sea bed has been predicted to consist of salt beds of pure magnesium sulfate (Kargel *et al.*, 2000 ▸; Spaun & Head, 2001 ▸) and could be derived from sinking MS7 and MS11. Depending on the heat flux coming from the core, deposited MS11 should subsequently dehydrate to MS7 (Prieto-Ballesteros & Kargel, 2005 ▸). However, even if MS11 forms simultaneously with or even just immediately after the ice, it will still be forming in a lower-viscosity brine than NMS16 (ρ = 1.623 g cm^−3^), which forms at a much lower temperature and which, despite its high density, would therefore have less opportunity for sedimentation or fluid transport. This could result in an enhancement of NMS16 at shallow depths within the Europan ice crust.

### 4.3. Relationship with surface morphologies and the observed non-ice component   

In this paper we have investigated the formation of hydrated salt species from Europa’s ocean water and the complex nature of the precipitates produced by a rapid-freeze experiment compared with a slow-freeze one. Differences between these could potentially infer likely associations with one or other of the various surface features summarized in Section 1.2, depending on differences in delivery mechanism and the associated rate of freezing.

Note, however, that once delivered to the surface or near-surface regions, freshly precipitated salts will be subjected to a number of destructive and modifying processes. For example, laboratory experiments have shown that, for typical Europan surface temperatures, the activation energies for the removal of H_2_O from MS7 and NS10 are such that MS7 should remain hydrated for ∼10^11^–10^14^ years, while NS10 will probably dehydrate on a scale of ∼10^3^–10^8^ years (McCord *et al.*, 2001 ▸).

Europa is also imbedded in an intense radiation environment, with its surface continually bombarded by energetic electrons, protons and heavy ions (Paranicas *et al.*, 2009 ▸), along with a lesser (∼2%) energy flux of solar UV photons capable of dissociating H_2_O. The dominant ionizing particles at the surface are electrons and protons, ranging in energy from <10 keV to >10 MeV, with average energies in the MeV range and penetration depths of the order of hundreds of micrometres. Extremely energetic electrons and bremsstrahlung X-rays will penetrate more deeply, while micrometeoroid impact gardening will simultaneously bury the radiation products and bring fresh material up from depth.

The radiolytic production/destruction rate is described by the *G* value, which is the number of molecules produced or destroyed per 100 eV of energy absorbed by a substance. For example, CO_2_ in H_2_O ice is destroyed at a rate *G*(−CO_2_) = 0.55 per 100 eV, while the production of SO_2_ from sulfate has a typical value of *G*(SO_2_) = 0.004 (Johnson *et al.*, 2004 ▸) (these are representative of both anhydrous and hydrated phases since production of O_2_ – a typical radiolysis product of water – is not observed experimentally; McCord *et al.*, 2001 ▸). Although *G*(SO_2_) is relatively small, over geological timescales, freshly exposed sulfates will nevertheless suffer radiation damage. Gardening models suggest that irradiated material could be vertically mixed to depths of up to 10 m (Carlson *et al.*, 2009 ▸).

SO_2_ was the second compound detected on Europa (Lane *et al.*, 1981 ▸), its 0.28 µm absorption feature being present only on Europa’s trailing side. The SO_2_ linearly correlates with Europan hydrate phases (Hendrix *et al.*, 2002 ▸, 2008 ▸) and is consistent with the radiolytic sulfate cycle on Europa, whereby newly formed SO_2_ is photolytically and radiolytically decomposed (Schriver-Mazzuoli *et al.*, 2003 ▸; Moore *et al.*, 2007 ▸) with a lifetime of a few years in the top 100 µm of the Europan surface (Moore *et al.*, 2007 ▸). The decomposition products reform sulfate in a repeating cycle, and a radiolytic equilibrium SO_2_ abundance is formed that is sensitive to the total sulfur-to-water ratio (Moore *et al.*, 2007 ▸). However, uniform outgassing of SO_2_ over the Europan surface is ruled out by the absence of SO_2_ on the leading side, which suggests, instead, that the SO_2_ on Europa could be mainly derived from implanted S ions from its neighbouring satellite Io [as originally suggested by Lane *et al.* (1981 ▸), though other endogenous sources such as inclusions, hydrates or clathrates have also been suggested]. Io is known to be a source of SO_2_, most of which quickly becomes dissociated in Jupiter’s magnetosphere, allowing a flow of S and O ions to be delivered to the Europan surface. However, since Jupiter’s magnetosphere rotates faster (∼10 hours) than the orbital period of Europa (∼3.6 days) these and other charged particles are carried over Europa from its trailing to leading hemisphere as the magnetosphere rotates over its body. Brown & Hand (2013 ▸) identified a spectroscopic feature on the trailing hemisphere of Europa which they attributed to Mg sulfate, proposing that, while the Mg is endogenous, the S is exogenous with the sulfate being produced by radiolytic processing. Similarly, deposits of sulfuric acid hydrates (H_2_SO_4_·*n*H_2_O) have been proposed to form on the Europan surface by radiolysis involving exogenous S (Dalton *et al.*, 2013 ▸).

The Europan surface is thus a complex dynamic environment subject to many physical processes [see *e.g.* the review by Carlson *et al.* (2009 ▸)], with highly modified non-ice components derived from both endogenous and exogenous matter. However, because of the differential in implantation rates between the leading and trailing hemispheres, only those areas of limited exogenic processing are likely to contain surface materials whose compositions provide a closer match to pristine oceanic precipitates (Dalton *et al.*, 2013 ▸).

Beneath the surface, however, salt precipitation from freezing Europan cryomagma may play a role in the formation of chaos terrain. Muñoz-Iglesias *et al.* (2014 ▸) investigated the precipitation of MS7 and MS11 alongside ice and CO_2_ gas clathrate hydrate as a function of MgSO_4_ concentration. The volume change associated with the formation of these phases was found to depend on the MgSO_4_ concentration and could potentially lead to the creation of chaos terrain either via the formation of surface fractures or via collapse, depending on the positive or negative volume change associated with the specific mix of sulfate, ice and clathrate. Presumably, corresponding effects will exist for the MEOS salts, other than MS7 and MS11, identified in the present work. Although clathrates have not yet been detected on Europa, their formation via the introduction of clathrate-forming gases into liquid saline water will remove water from the solution, resulting in increased brine concentration. If this was to occur inside an aqueous cryo-magmatic chamber, or sill, within the ice crust, the clathrates would separate from the remaining brine by either sinking or floating, depending on the specific temperature and salinity (Safi *et al.*, 2017 ▸) which will be determined by temperature-dependent salt precipitation. If clathrates close to the surface were to dissociate, the very rapid release of gas and large negative change in volume would probably cause fracturing and gravitational collapse and could therefore also be a contributory process to the formation of chaos terrain.

### 4.4. Implications for life on Europa   

Life on Earth often appears to thrive at the edges and interfaces between different environments. These are places of disequilibrium and the surface–ice–ocean system on Europa should represent a prime planetary example of a global interface environment. However, life is also constrained by access to resources and environmental requirements, such that the geographical distribution of any life forms that currently exist (or might have existed in the past) on Europa is likely to be heterogeneous. Lipps & Rieboldt (2005 ▸) identified ∼15 broad categories of habitat that could be possible on Europa, including locations on the sea-floor, in the water column and within the ice crust itself. Ocean circulation, geological activity and thermal history will have resulted in global salt transport (Travis *et al.*, 2012 ▸), such that habitable environments on Europa are likely to be predominantly saline in nature.

**Figure 4:**
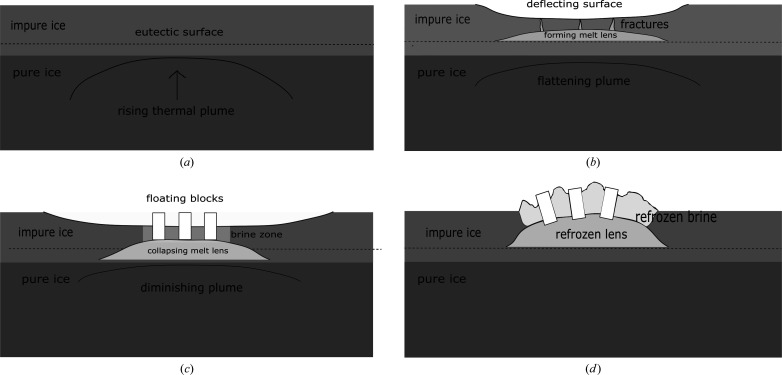
Origin of Europan surface morphologies III: near-surface melt water during chaos terrain formation. (*a*) Ascending thermal plume in the subsurface approaches the pressure-melting eutectic point of the overlying impure brittle ice; (*b*) melting causes surface subsidence that hydraulically confines water and produces tensile cracks; (*c*) hydro-fracture from the melt lens calves ice blocks, while fracture and brine infiltration form a granular matrix; (*d*) refreezing of the melt lens and freezing of the now brine-rich matrix raises the chaos feature above the surrounding terrain, and can cause domes to form between blocks and at the margins. Figures after Schmidt *et al.* (2011 ▸).

**Figure 5:**
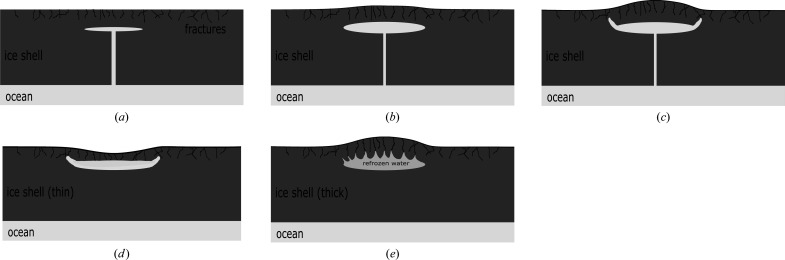
Origin of Europan surface morphologies IV: evolution of a subsurface saucer-shaped sill and its surface expression, creating pits, domes or small chaos. Upward intrusions into the surrounding ice by freezing water from a sill (*a*) lead to surface disruption or extrusion [(*b*) and (*c*)], to form spots with a subsurface sill [(*d*) or (*e*)]. Figures after Manga & Michaut (2017 ▸).

On Earth, channels and inclusions within sea ice containing liquid brines host a wide range of sympagic organisms (bacteria, microalgae, viruses, fungi, protozoans and metazoans) within a physicochemical environment subject to oscillating gradients in temperature, salinity, pH, dissolved inorganic nutrients, dissolved gas and light signatures (Mock & Thomas, 2005 ▸). Osmotic conditions within terrestrial brine channels are controlled by brine salinity and are therefore defined by temperature-dependent salt precipitation (*e.g.* the precipitation of NS10 below 266.75 K; Butler *et al.*, 2016 ▸). Microbes inhabiting brine channels near the upper surface of the ice can experience saline concentrations >20% (Kottmeier & Sullivan, 1988 ▸; Arrigo & Sullivan, 1992 ▸; Mock, 2002 ▸), while the onset of ice melt can very quickly expose them to freshwater lenses with 0% salinity (Thomas & Dieckmann, 2002 ▸). Although the solar flux at Europa is ∼27 times weaker than that at Earth, light penetration into the Europan ice crust could be up to a few metres so could in principle support an analogous ice–brine ecosystem (Martin & McMinn, 2018 ▸), where life-sustaining nutrients and fuel could be supplied from the surface (via radiatively formed oxidizing molecules; Johnson *et al.*, 2004 ▸; Paranicas *et al.*, 2009 ▸) and/or sea-floor processes (Hand *et al.*, 2009 ▸). Periodic reductions in salinity, in keeping with those on Earth, could be provided by the formation of melt water lenses (*e.g.* Fig. 4 ▸). Furthermore, although the surface of Europa is both young and active in geological terms, it is likely to remain stable for tens of thousands of years (Greenberg, 2008 ▸), providing a potentially long-lived ecological niche within the ice crust in which the temperature-dependent salt precipitates identified in the slow-freeze experiment could, conceivably, play similar regulatory environment-controlling roles. Although temperatures towards the surface on Europa can reach as low as ∼50–90 K, the lower limit of ∼77 K for microbial activity seen in laboratory cultures on Earth (Junge *et al.*, 2006 ▸) falls within this range (although at the lowest temperatures this may represent cell maintenance for survival rather than growth), with warmer conditions existing closer to the ice–ocean interface. As a stable low-temperature phase, with a possible enhancement at shallow depths, the nucleation of NMS16 could prove to be of particular astrobiological significance as an environmental regulator.

The biological pump on Earth is the global mechanism by which organic carbon is delivered to the ocean interior and sea-floor via the sinking of particulate organic matter, largely derived from deceased planktonic organisms. Sinking particles often coalesce to form aggregates (the marine snow), which increases their sinking velocity and is largely determined, via equation (2), by their size and excess density (Smayda, 1971 ▸; De La Rocha & Passow, 2007 ▸) (though the interplay between aggregate radius and density is not straightforward; *e.g.* Passow & De La Rocha, 2006 ▸). On Earth, sinking velocity plays a significant role in (*a*) the long-term sequestration rate of atmospheric carbon, once it has been fixed by organisms in the photic zone, and (*b*) the delivery of organic nutrients at depth. Sinking velocity, and therefore achieved depth, is enhanced by the incorporation of mineral ballast materials (Ploug, Iversen & Fischer, 2008 ▸; Ploug, Iversen, Koski & Buitenhuis, 2008 ▸), which have themselves been found to promote coagulation of organic particles (Avnimelech *et al.*, 1982 ▸; Beaulieu *et al.*, 2005 ▸; Verspagen *et al.*, 2006 ▸; Passow *et al.*, 2014 ▸). Although calcium carbonate biominerals are a ready source of ballast, the primary non-biogenic source is lithogenic dusts and clays supplied by atmospheric deposition and river inflow, the availability and composition of which vary both locally and globally (as well as historically). Since sulfates (including Mg and Ca sulfates) are widely used, albeit as solutes, as flocculant additives in water treatment and various commercial/industrial processes, we speculate as to whether low-temperature salt precipitates could assume the role of lithogenic matter and similarly promote the coagulation of any organics released from possible ice–brine habitats on Europa. Of relevance here is the recent discovery of abundant gypsum (CS2) crystals embedded within aggregates of Phaeocystis algae collected throughout the water column and sea-floor at depths below 2 km in the ice-covered Arctic Ocean. These are probably a significant, previously unrecognized, contributor to the biological pump in cold regions (Wollenburg *et al.*, 2018 ▸, 2020 ▸). The CS2 was shown to have been precipitated within sea ice and subsequently released into the water column during melting. Although CS2 is one of the highest-density precipitates, it appears to be only a minor component in our MEOS experiments. Considering how MEOS composition differs from terrestrial sea water, other precipitated phases could potentially play a similar role in the transfer of organic matter within the Europan ocean. Given the weaker gravitational acceleration on Europa, the more dense mixed Na–Mg phases (*e.g.* NMS4, NMS5 and NMS15; see Table 1 ▸ and the discussion in Section 4.1) may be more important, even if less abundant.

The above presupposes that life is already somehow established on Europa. However, salts may also have been important in the formation and development of the necessary prebiotic components prior to any potential beginnings of Europan life. On the early Earth the concentration of prebiotic organic compounds in a global ocean was likely to have been extremely low (estimates range from ∼4 × 10^−3^ to ∼4 × 10^−12^ *M*; Stribling & Miller, 1987 ▸; Lahav & Chang, 1976 ▸; Miyakawa *et al.*, 2006 ▸) and therefore would have been too dilute for the synthesis of nucleotide bases and amino acids to compete with their decomposition by hydrolysis (Miyakawa *et al.*, 2006 ▸). However, the concentration within sea ice brine inclusions becomes orders of magnitude higher and the water activity within the inclusions so low that prebiotic molecular polymerization is favoured over hydrolysis (Miyakawa *et al.*, 2006 ▸). Although Europa is comparable to the Moon in size, its ocean volume is two to three times that of Earth, suggesting probable low organic concentrations, as per the early Earth. This suggests that brines within the ice crust could potentially play a similar role in building complex biomolecules. In addition, the likely thermal structure of the crust suggests that there should be a shallow region (*i.e.* at a depth of a few kilometres) that is favourable for the polymerization of biomolecules, since at low temperatures the Gibbs energies for biomolecule polymerization become negative, allowing for spontaneous polymerization (Kimura & Kitadai, 2015 ▸). This may be further enhanced by the presence of saline species. Glycine (Gly) polymerization has been shown (albeit in thermal dehydrating conditions) to be accelerated in the presence of MgSO_4_ (Kitadai *et al.*, 2011 ▸). In these experiments up to 6-mer of Gly polymers were synthesized with a total yield ∼200 times greater in the presence of MgSO_4_ than from Gly on its own. Mg^2+^ ions have also been found to stabilize nucleic acid duplexes to a greater extent than the same concentration of Na^+^ ions (Williams *et al.*, 1989 ▸).

In the context of the ‘RNA world first’ hypothesis for the origin of life, following similar arguments, cold ice–brine environments on Earth may have been an essential low-temperature step in the early replication of nucleic acids (Trinks *et al.*, 2005 ▸; Price, 2007 ▸; Vincent *et al.*, 2004 ▸; Feller, 2017 ▸) and could play a similar role on Europa. Minerals can provide solid surfaces for a range of biogenic interactions (Cleaves *et al.*, 2012 ▸), and experiments show that nucleotide oligomerization can be catalysed, for example, by clay surfaces (Ferris, 2002 ▸; Huang & Ferris, 2006 ▸). When NaCl or MgCl_2_ are added, both cause an increase in oligomer length, but by different amounts (Jheeta & Joshi, 2014 ▸). This last point could raise the possibility of a potentially different biochemical basis for any life that may have developed on Europa, compared with Earth, based on differences in the available inventory of salt phases. Interestingly, the two main precipitates observed in this study are Na and Mg sulfate phases of high hydration state, but with MS11 ≃ 2 × NS10. As discussed in Section 4.2, stratification into Na-rich salts near the surface and Mg-rich salts below could either restrict life-producing biogenic reactions to the near-surface regions, in keeping with the Na-rich hydrosphere of Earth, or possibly result in two distinct bio-geochemical near-surface and deep subsurface zones.

### 4.5. Future work   

#### 4.5.1. *In situ* fast freezing on I11   

The freezing rates associated with the various mechanisms for delivering oceanic matter to the Europan surface (Section 1.2) are likely to cover a range that exceeds the rapid- and slow-freezing experiments reported here. In our fast-freeze experiment we began cooling the MEOS sample from a starting temperature of 256 K. However, it is possible that employing varying rates of cooling and starting temperatures could result in the freezing-in of different molecular liquid arrangements which could influence precipitate formation and subsequent evolution, as potentially could small local deviations in the chemical composition. Mechanisms involving very steep temperature gradients are also likely to subject their materials to fast or flash freezing. For oceanic water erupted by plumes, whose frozen contents return to the surface, the assemblage of salts could be even more complex than those reported here. Water droplets comprising part of a plume are frozen extremely quickly, with the loss of about 17% of the water by evaporation and sublimation if the water is originally liquid and at 273 K. The 17% loss of water corresponds to a 20% increase in the concentration of non-volatile solutes (Pasek, 2020 ▸).

In the laboratory, flash freezing can be achieved by dropping solutions onto a cold surface or into liquid nitrogen. This is likely to produce a disequilibrium solid whose initial state (*i.e.* phase structure and amorphicity) will be very different from either the rapidly or slowly cooled solutions. For example, Fortes (2018 ▸) notes that immersion of pure water in liquid nitrogen probably produces stacking-disordered ice. This contains a mixture of strained cubic and hexagonal stacking sequences, rather than just the hexagonal sequence produced by ‘normal’ cooling, and is also formed when water is vapour deposited, or frozen extremely fast, as submicrometre-sized droplets. In addition, *ex situ* formation necessarily requires *ex situ* preparation, which for X-ray powder diffraction means grinding, loading into capillaries, transportation and mounting on the instrument. Under such conditions some degree of relaxation, crystallization or phase transformation could occur. Indeed, as Figs. 10 ▸–12 ▸ ▸ show, under rapid cooling, significant changes occur within the first one or two temperature steps. Such changes may occur even more quickly for a disequilibrium solid formed by flash freezing.

**Figure 6:**
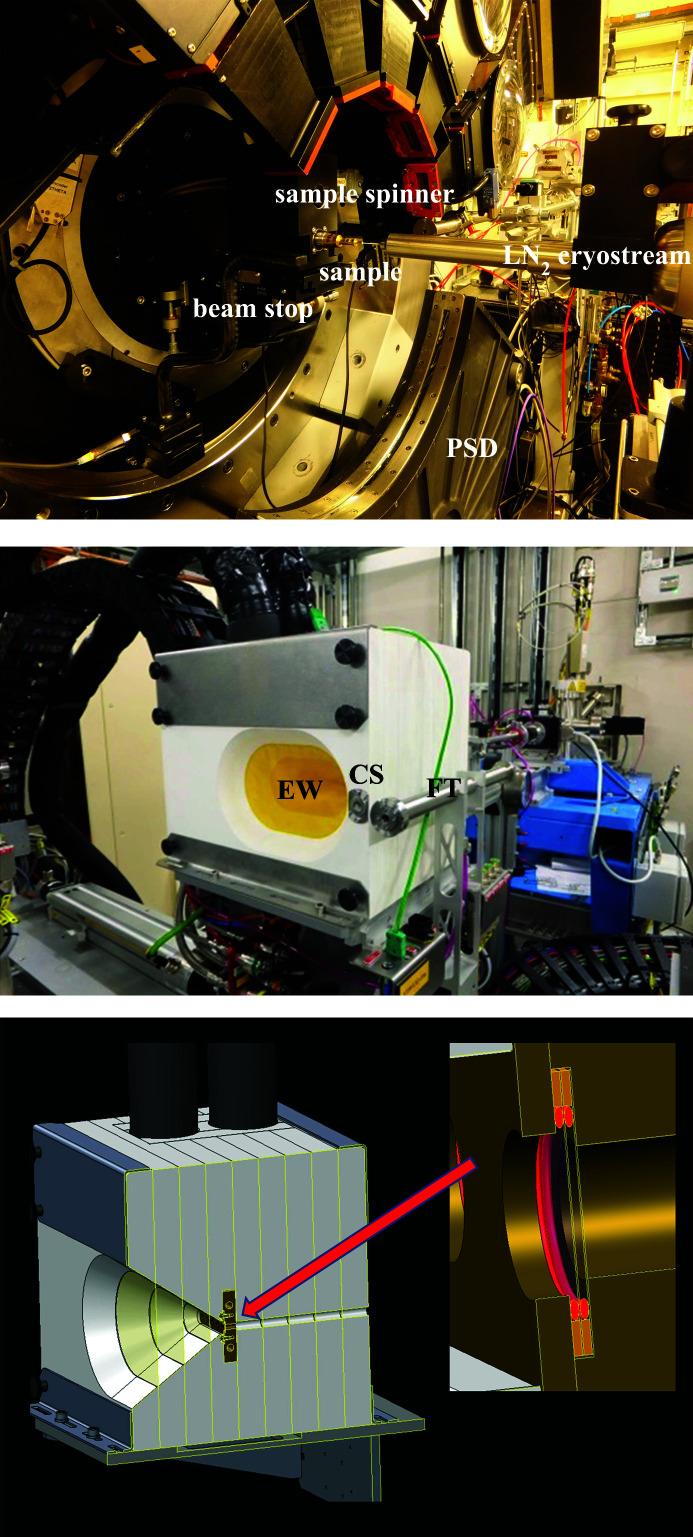
Experimental apparatus. (Top) *In situ* fast-freeze measurements; view is towards the incident X-ray beam, showing the sample capillary, the orientation of the liquid nitrogen (LN_2_) cryostream and the position sensitive detector (PSD). (Middle) Slow *in situ* cooling rate cold cell installed in the I11 Long-Duration Experiments facility; view is towards the incident beam direction and shows the large exit window (EW), calibration standard (CS) housing and X-ray flight tube (FT) for when other long-duration cells are being measured. (Bottom) Schematic showing the cold cell’s interior sample chamber formed by diamond windows held between o-rings and mounted in a cooled copper block, located within the large insulated housing. [Full details given by Thompson *et al.* (2018 ▸).]

**Figure 7:**
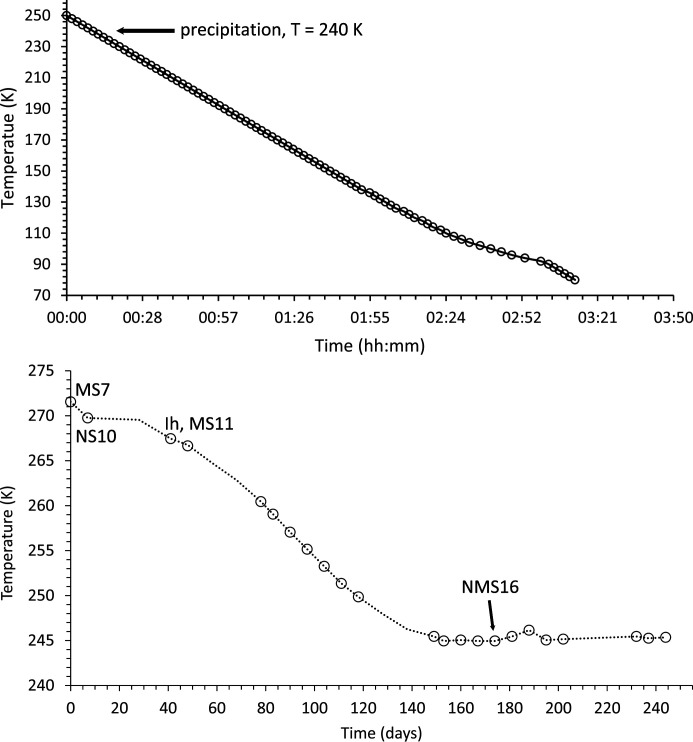
*In situ* time–temperature profiles for (top) fast- and (bottom) slow-cooling experiments. In both plots the open circles represent the points at which diffraction data were collected. In the bottom panel the dotted line represents the temperature profile of the cell based on the temperature recorded at the start of each day. The short plateaux after the second diffraction point is due to a networking failure early in the experiment such that the cell remained at constant temperature. The gaps between diffraction data points at 20, 60, 130 and 220 days are due to scheduled synchrotron shutdowns. The small rise in temperature at ∼190 days is due to ice build-up in the chiller reservoir which restricted coolant circulation until being removed.

**Figure 8:**
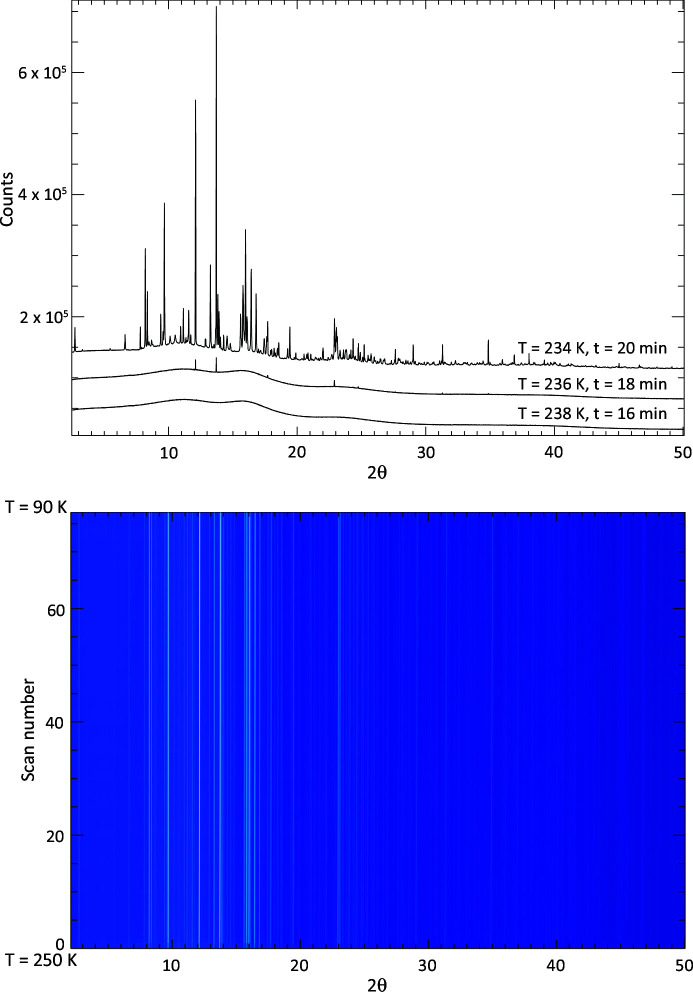
(Top) Successive synchrotron X-ray powder diffraction patterns showing crystallization of the MEOS under fast-cooling conditions. Data collected at 15 keV (0.824603 Å); patterns are offset for clarity. The bottom trace is the liquid phase at a temperature of 238 K, reached 16 min after the start of the cooling ramp; the middle trace shows formation of the hexagonal ice phase 2 min later at 236 K; this is followed after another 2 min by the crystallization of multiple hydrated phases at 234 K, shown by the top trace. (Bottom) Waterfall-type plot of diffraction patterns, showing consistency of the phase assemblage once formed. Note, owing to colour map stretching to show both weak and strong features, relative variations in intensity for individual peaks are not visible.

**Figure 9:**
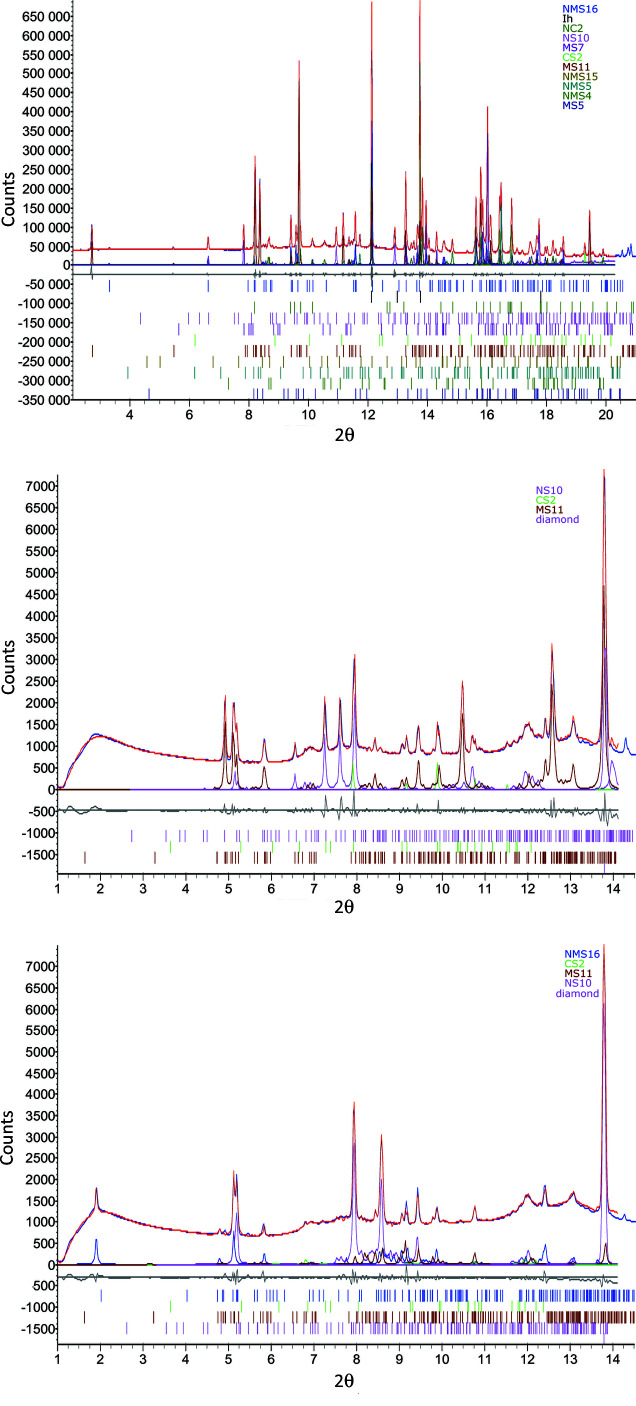
Examples of fits (red line) to XRD data (blue), along with individual phase contributions, obtained for (top) fast- and (middle and bottom) slow-cooling-rate experiments. The two slow-cooling-rate patterns were collected two weeks apart on days 167 and 181, respectively, of the long-duration experiment and show the development of NMS16 via the Bragg peak at ∼2° 2θ, as also indicated in Fig. 7 ▸. Data collected at 15 keV (0.824603 Å) and 25 keV (0.49388 Å, average) for the fast- and slow-cooling-rate experiments, respectively

**Figure 10:**
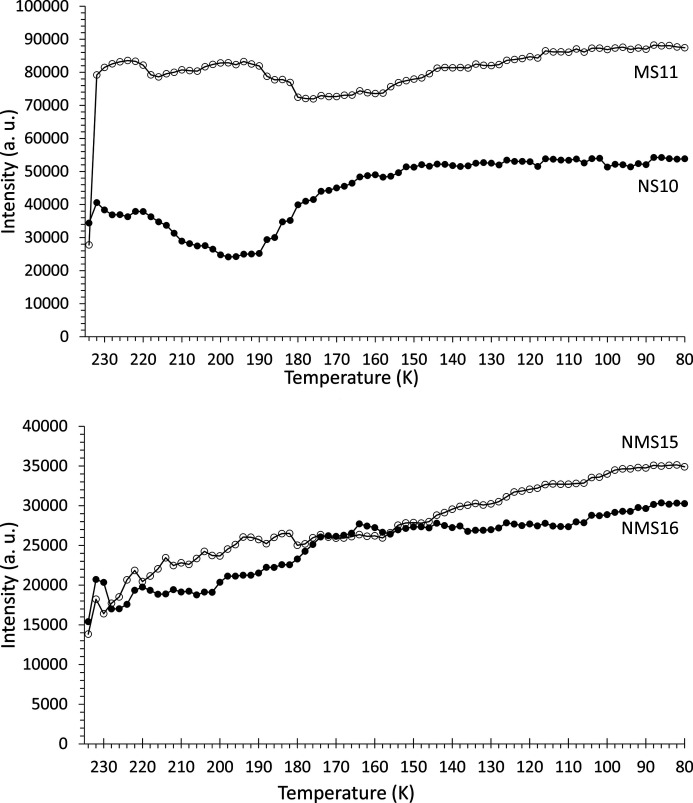
Rapid cooling I: total scattered intensity for each precipitated phase at each temperature step for (top) main phases MS11 and NS10 and (bottom) NMS15 and NMS16.

**Figure 11:**
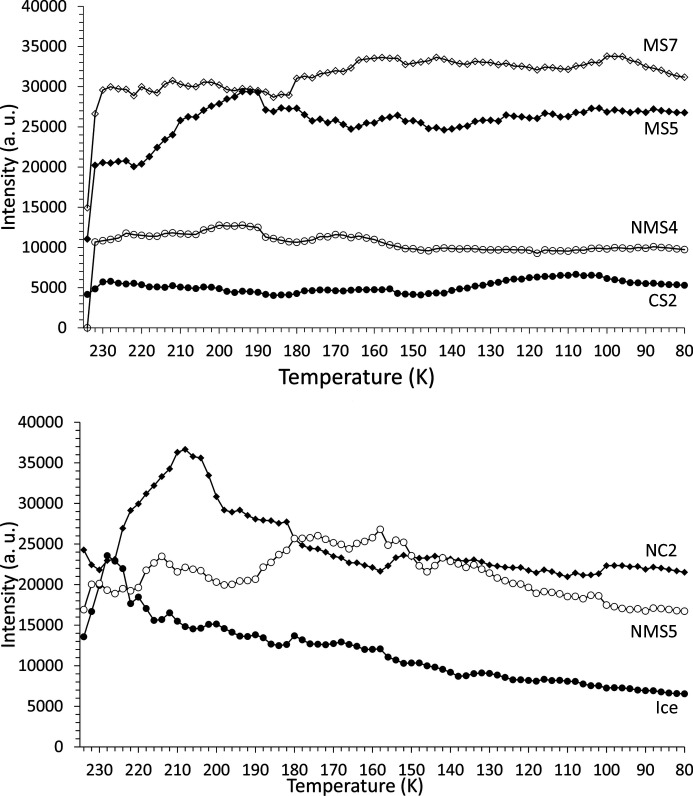
Rapid cooling II: total scattered intensity for each precipitated phase at each temperature step for (top) MS7, MS5, NMS4 and CS2, and (bottom) NC2, NMS5 and ice.

**Figure 12:**
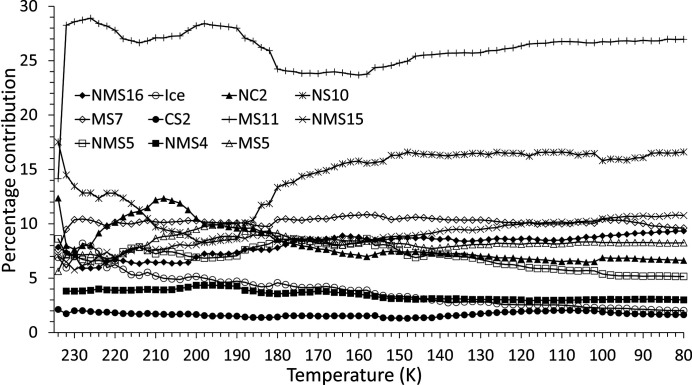
Rapid cooling: the proportional percentage contribution of each phase to the scattered intensity as a function of temperature.

**Figure 13:**
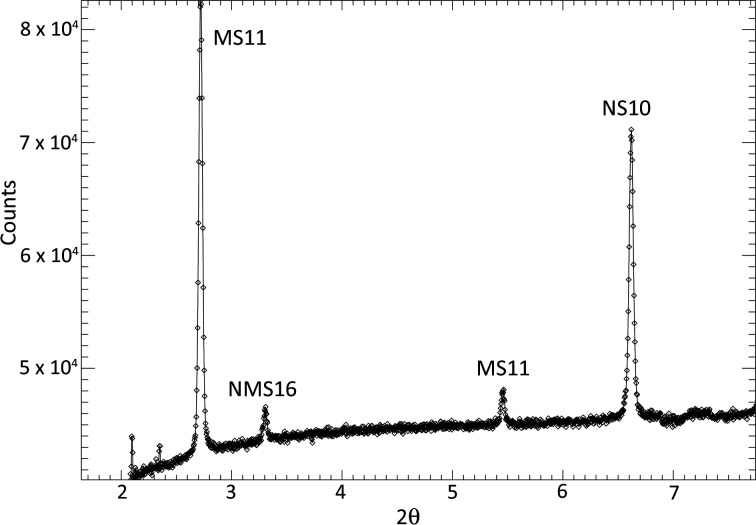
The 001 peaks of the highly hydrated salt phases NMS16 [Na_2_Mg(SO_4_)_2_·16H_2_O], MS11 (MgSO_4_·11H_2_O, meridianiite) and NS10 (Na_2_SO_4_·10H_2_O, mirabilite). The peak at 5.446° 2θ is the MS11 002 peak. The two peaks at 2.098 and 2.349° 2θ are unidentified. Data collected at 15 keV (0.824603 Å).

**Figure 14:**
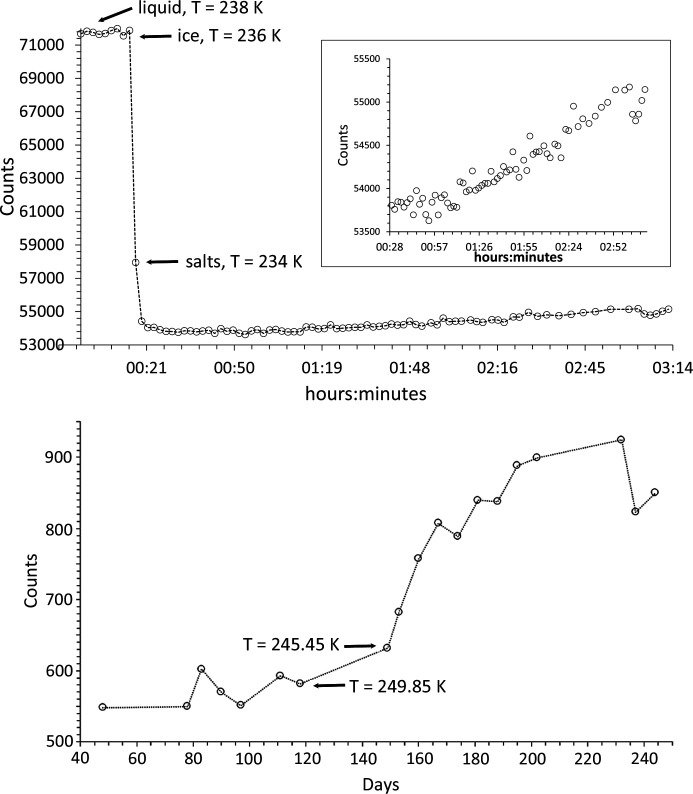
Changes in diffuse scattering background for the rapid-cooling experiment, showing (top) change from liquid to ice to the precipitation point of salt phases. After an hour, at ∼175 K, there is an increase (inset) in diffuse scatter due to an increase in static disorder at low temperature related to increased rigidity of the bonded water molecules in the salt phases. (Bottom) Slowly cooled experiment. The rise in diffuse scatter occurs between 120 and 150 days at or near base temperature for the cell (245 K). This increase is attributed to an amorphous phase arising from the freezing of liquid water at low temperatures, as a result of a reduction in salinity due to precipitation of salt phases. See discussion in Section 3.2.

However, the existing beamline setup can be used for *in situ* flash freezing. As Fig. 6 ▸ shows, the capillary is normally situated inside the cryostream nozzle. The cryostream is mounted on a separate large motorized xyz table separate from the diffractometer, allowing the cryostream to be pre-set to a chosen temperature and driven over the capillary. Although there may be a small thermal lag due to the thermal conductivity of the capillary, the PSD can be set to collect data before, during and after freezing, returning data on phase evolution.

Although the current PSD is relatively fast at ∼20 Hz frame rate (Thompson *et al.*, 2011 ▸), in normal use it is limited by a combination of data collection count time (two exposures separated by a 0.25° 2θ step), readout speed and network file storage write times, which limit the practical temporal resolution to ∼3–5 s (although this can be improved by using local storage and/or fast triggering at fixed position). At the time of writing, the I11 beamline is undergoing an extensive upgrade programme to the monochromator (completed), diffractometer (completed), source (due mid-2022) and detectors (early 2022). The last will see the existing Mythen-II-based PSD replaced with one based on a 2D tiled design using Mythen-III technology (Andrä *et al.*, 2019 ▸). This will eliminate two-position scanning and provide a significant gain in readout speed, lower background noise and shorter dead time at high photon rates, allowing for frame rates in the kHz range for *in situ* flash-freezing measurements.

#### 4.5.2. Application to other planetary objects   

Many of the icy objects in the outer Solar System are believed to harbour liquid oceans, as suggested variously by observation, modelling or plausible circumstance [see *e.g.* reviews by Hussmann *et al.* (2006 ▸), Massé *et al.* (2014 ▸) and Nimmo & Pappalardo (2016 ▸); see also Table 1 of Thompson *et al.* (2018 ▸)]. Some of these will be in direct contact with their rocky cores. For example, gravimetric measurements for Enceladus suggest rock–ocean and ice–ocean interfaces at depths of ∼50 km (∼5.3 MPa) and 35–40 km (3.6–4.2 MPa), respectively (Iess *et al.*, 2014 ▸), and with pressures well within the range experienced by terrestrial life. For other objects the ocean will be isolated by high- and low-pressure ice phases. For example, Europa’s neighbour Ganymede is the largest moon of the Solar System and has a surface containing both old, densely cratered terrain and widespread tectonically resurfaced (*i.e.* younger) regions similar to Europa. Its interior has a differentiated structure (Sotin & Tobie, 2004 ▸) and is also subject to tidal heating due to resonant-forcing of its orbital eccentricity. However, because of its larger size and higher abundance of water, relative to its denser mantle materials, pressures at the water–rock interface are likely to be as high as 1.2 GPa. Consequently, any liquid ocean will probably be sandwiched between layers of ice I at the surface and ice V and VI at the sea bed (Sotin & Tobie, 2004 ▸). Such high pressures are likely to close microfractures in the high-pressure ice, inhibiting significant water–rock interaction post-differentiation (Vance *et al.*, 2007 ▸). This means that the ocean composition will have been determined largely by the extent of the water–rock interaction during the period of differentiation and probably preserved by the ocean’s subsequent isolation. Owing to the high bulk-water-to-rock ratio and reduced hydrogen fugacity caused by the loss of hydrogen to space, primordial water–rock interactions within chondritic parent accretion materials would, in the case of Ganymede, have favoured a rise in the solubility of sulfate and Mg^2+^ (King *et al.*, 2004 ▸; Zolotov & Kargel, 2009 ▸), resulting in an ocean dominated by MgSO_4_. The actual salinity of Ganymede’s ocean is not known, but it could be 3–10% (Vance *et al.*, 2014 ▸), while reflectance spectra measured by the Galileo mission suggest that the predominant non-ice surface materials are moderately hydrated materials similar to those on Europa, *i.e.* salts (McCord *et al.*, 2001 ▸).

Beyond the ocean worlds of the Solar System there are over 4000 known exoplanets, many of which have masses and radii comparable to Earth’s. Theoretical analysis suggests that ∼26% could be ocean worlds (Quick *et al.*, 2020 ▸), a proportion of which could reasonably be assumed to be similar to the icy moons of our own Solar System. This is a highly active field of academic research of obvious significance, where observational techniques and analyses are rapidly evolving. Atmospheric spectroscopy of exoplanets is now routinely possible using current observational techniques and instrumentation and existing data sets (*e.g.* Seager & Deming, 2010 ▸; Seager, 2013 ▸; Lacour *et al.*, 2019 ▸; Tsiaras *et al.*, 2019 ▸) and will improve with the next generation of space telescopes (*e.g.* the James Webb Space Telescope scheduled to launch in 2021) and future Earth- and space-based instruments currently being designed (*e.g.* ESA’s Atmospheric Remote-Sensing Infrared Exoplanet Large Survey – ARIEL – mission due to launch in 2028). Atmospheric compositional data from these instruments will feed into climate and planetary models with the expectation of identifying habitable worlds (*e.g.* Catling *et al.*, 2018 ▸; Kaltenegger *et al.*, 2020 ▸). For example, the TRAPPIST-1 system comprises seven Earth-sized planets labelled, in order of radial distance, TRAPPIST-1 b through h. Of these, TRAPPIST-1 e, f and g all orbit within the ‘habitable zone’ where water could potentially exist in liquid form. Current models (Lincowski *et al.*, 2018 ▸) suggest TRAPPIST-1 e could host liquid water, while TRAPPIST-1 f and g could be frozen icy worlds, depending on their original water budget. The central star of the TRAPPIST-1 system is an M-type dwarf star. These are smaller than the Sun in both mass and size, but are in fact the most abundant type of star (Henry *et al.*, 2006 ▸) with high occurrences in multi-planet systems (Ballard & Johnson, 2016 ▸; Gillon *et al.*, 2017 ▸) and habitable-zone Earth-sized planets (Dressing & Charbonneau, 2015 ▸), making the existence of icy ocean exo-worlds highly probable.

Given the extremely widespread production from stellar sources, and distribution throughout interstellar space, of silicate- and carbonaceous-based cosmic dust grains as the ultimate precursor planetary material (*e.g.* Jones, 2007 ▸; Blum & Wurm, 2008 ▸) it is likely that a chondrite-like rocky core will lie at the heart of exo-ocean worlds and that water–rock interactions will result in the existence of saline exo-oceans.

The geological record shows that Earth experienced a number of freezing episodes 630–720 Ma ago, during the Neoproterozoic eon. At this time extensive glaciation covered much or all of the oceans, separating them from the atmosphere and probably resulting in the large-scale extinction of preglacial microorganisms and plants [see *e.g.* the review by Banik (2016 ▸)]. These Snowball Earth episodes (the Cryogenian) were the result of runaway ice–albedo feedback. The albedo was further increased by an extensive MS10 salt crust in tropical regions, formed by the sublimation of sea ice, the MS10 having precipitated within the sea ice once temperatures fell below 150 K (Carns *et al.*, 2015 ▸, 2016 ▸). It was during the Cryogenian that eukaryote cells first acquired the ability to biosynthesize certain sterols (C_26_–C_30_; Hoshino *et al.*, 2017 ▸). Synthesis of C_29_ sterols, for example, significantly reduces the temperature dependence of membrane dynamics, extending the temperature range for biological membrane processes. This confers a distinct evolutionary advantage against large temperature fluctuations and probably gave rise to the global dominance of green algae within the marine ecosystem. The fossil record also shows that the end of the Cryogenian coincided with the rise of predation by heterotrophic plankton (van Maldegem *et al.*, 2019 ▸). These are marine microbes that rely on gaining energy by the consumption of other organisms (*e.g.* the green algae). The rise of predation established a food chain and ultimately led to the Cambrian explosion and the development of intricate life forms, including the lineages from which all animals, including humans, derive. Despite the initial extinction, the Cryogenian period was therefore a driver for evolution rather than an inhibitor. The conditions under which an exoplanet orbiting within the habitable zone with liquid water covering a proportion of its surface can enter (and recover from) a snowball phase are a matter of current debate. However, they will depend on the spectral type of the star, the rotational and orbital properties of the planet, and its geographical characteristics (*e.g.* Checlair *et al.*, 2017 ▸, 2019 ▸; Foley, 2019 ▸; Paradise *et al.*, 2019 ▸; Walsh *et al.*, 2019 ▸; Yue & Yang, 2020 ▸), while the development and nature of a salt crust will depend on the details of the ocean composition. The abiotic origin of life is arguably the greatest unsolved scientific problem, but thus far has no standard model. Thus, if current theories on the role of low-temperature salt precipitates in the origin and development of life (as discussed in Section 4.4) are correct, then icy or frozen ocean worlds could represent a universal step in the development of life throughout the universe.

In this paper we have focused on a single ocean composition based on a chondritic composition. However, ‘the’ chondritic composition is largely a notional average based on abundances deduced from primitive meteorites, whose localized elemental abundances were determined by those of the solar nebula. It is generally held that the bulk of the matter in the primordial solar nebula was relatively uniform in chemical and isotopic composition (Palme *et al.*, 2014 ▸). Furthermore, the elemental composition is roughly similar to that of other stars in the solar neighbourhood. However, there are observed compositional differences between stars at similar evolutionary stages (*e.g.* dwarf stars). These are not caused by internal properties (*i.e.* nucleosynthesis) but reflect the variation in the chemical evolution of the Galaxy, whose elemental and isotopic composition has evolved with time (Timmes *et al.*, 1995 ▸). Elemental production by nucleosynthesis in successive generations of stars leads to heavy-element (*i.e.* elements beyond C, N and O) enrichment of the interstellar medium over time. Hence there is an overall increase in the ‘metallicity’ of both the Galaxy and newly forming stars, which similarly inherit the elemental abundances present at the place and time they form. Their planets, in turn, inherit the materials left over from the star-forming process, which will have been processed to varying extents during both the stellar formation and planetary build. The process is stochastic on a local scale, but there is a gradient in the Galaxy with central, denser regions being more chemically evolved (Anders *et al.*, 2017 ▸) owing to faster star-formation rates. The lifetime of a star is determined by its initial mass, ranging from many billions of years for an M-type star to only a few million years for stellar masses ∼10× greater than an M-type star. The orbit of a star within the galactic disc will also tend to wander outwards with time because of the phenomenon of radial migration, which is influenced by past galactic collisions, the number of spiral arms, and the size and movement of the central galactic bar (Minchev & Famaey, 2010 ▸; Minchev *et al.*, 2018 ▸). Since dust production mostly occurs towards the end of a star’s life, those that are dying and producing dust at any one time and place may have quite different ages, formation sites and initial compositions. Isotopic analysis of unprocessed inorganic pre-solar grains identified within chondrite meteorites suggests their formation around a number of distinct stellar sources: red giant/asymptotic giant branch (RGB/AGB) stars, supernovae, and, possibly, novae and Wolf–Rayet stars (*e.g.* Leitner *et al.*, 2012 ▸; Nguyen *et al.*, 2016 ▸). The range in isotopic composition suggests that a minimum of 35–40 stellar sources contributed to the nebula from which our Sun and Solar System formed (Alexander, 2001 ▸), while its birth place was approximately 2000 light years closer in towards the galactic centre (Minchev *et al.*, 2018 ▸). Variation and fluctuations in the average chondritic composition over galactic length scales is therefore likely.

Clearly, ocean worlds, not only those of the Solar System but also the probable subset of the exoplanet population, are likely to have oceans that vary in their compositions, depending on their size (extent of water–rock interaction), specific ‘chondritic’ building block materials and position in their own nebula. This last point will determine volatile content which could also influence ocean composition. For example, Saturn’s moon Titan probably formed at a point in the Saturnian sub-nebula where temperatures were below the condensation temperatures of various primordial volatile species, such that concentrated eutectic solutions at temperatures below 273 K would have formed that were typically rich in CH_3_OH, NH_3_, CO_2_ and H_2_S (Sohl *et al.*, 2010 ▸). Under freezing conditions these would have been further concentrated and capable of facilitating the dissolution of mineral phases and the chemical reactions necessary to yield a saline ocean. In particular, the interaction of ammonia-rich liquids with magnesium sulfate, contained within chondritic core materials, could result in an ocean enriched in ammonium sulfate (Fortes *et al.*, 2007 ▸) rather than magnesium sulfate or sodium chloride. In exoplanetary systems, Titan-like planets could prove to be highly abundant since the effective temperature of Titan corresponds to that experienced by a body orbiting at 1 astronomical unit (Earth–Sun distance) around a late M-type dwarf star. Much, therefore, could be gained by applying the *in situ* experimental procedures, high-resolution time- and temperature-resolved synchrotron X-ray diffraction measurements, and crystallographic analysis of the present work to a systematic range of ocean compositions.

#### 4.5.3. Organic–inorganic interactions   

Finally, work has also begun on using the apparatus and procedures described here to investigate the role and behaviour of organics in precipitating brines. Amino acids, for example, are highly soluble and can be crystallized from aqueous solutions. They are also a well documented constituent of primitive carbonaceous chondrite meteorites and constitute a major organic component of the insoluble macromolecular material contained within these objects, reflecting the diversity of conditions that existed on their original parent bodies (Elsila *et al.*, 2016 ▸). The most abundant amino acid across all meteorites is Gly, which has also been identified in samples returned from comet 81P/Wild 2 by NASA’s STARDUST mission (Elsila *et al.*, 2009 ▸). Its cometary origin was confirmed by ^13^C isotope signature. Gly has subsequently also been detected in the coma of 67P/Churyumov–Gerasimenko by the ROSINA mass spectrometer on the Rosetta Orbiter (Altwegg *et al.*, 2016 ▸), while Gly precursor molecules have been identified in the astronomical spectra of many other comets including Halley, Hyakutake, Tempel-1, Giacobini–Zinner, Hartley 2 and Hale–Bopp. Hypervelocity impact experiments using precursor molecules within ice targets have also produced a range of amino acids, with Gly being the most abundant (Martins *et al.*, 2013 ▸). The presence of amino acids, and Gly in particular, on ice–ocean bodies (including the early Earth) is thus widely acknowledged to be likely, and would derive from either the original chondritic core, delivery via meteorites and impacts from asteroids (meteorite parent bodies) or comets, or *in situ* synthesis during impact.

Gly has three ambient-pressure polymorphs (α form, *P*2_1_/*n*; β form, *P*2_1_; γ form, *P*_2_), all of which can be crystallized from aqueous solutions (Surovtsev *et al.*, 2012 ▸). As with mineral phases, polymorph crystallization is a result of the complex interplay between kinetic and thermodynamic factors, *i.e.* the nucleation rate, the overall rate of crystal growth and the difference in the growth of different crystal faces. Studies of the crystallization outcomes in confined environments, at specific surfaces, on spray-drying, flash freezing or adding antisolvent (acetone, ethanol, methanol *etc*.) have been shown to preferentially select one or other Gly polymorph (Surovtsev *et al.*, 2012 ▸). Additionally, flash freezing of aqueous Gly solutions produces an amorphous phase, which transforms on heating to a fourth intermediary phase, prior to forming β-Gly. To our knowledge, no systematic studies of Gly crystallization in freezing saline environments have been performed. Since Gly is capable of incorporating into inorganic hydrated mineral structures (Kavitha & Mahadevan, 2013 ▸), such studies could provide new insights into the nature and origin of the non-ice surface mineralogy of icy bodies as well as the early prebiotic stages involved in the origin of life as discussed in Section 4.4.

## 5. Conclusions   

Europa has several parallels with Earth in that it is differentiated, has tectonic activity and has liquid water in the form of a global saline ocean. Although the ocean is covered by a relatively thick ice crust, various physical mechanisms, possibly acting at different times in the geologically recent history of the moon, are likely to have delivered significant quantities of oceanic matter to the surface, resulting in the presence of hydrated salt deposits. We have conducted experiments to identify the types of salts that may form and be present on the surface by investigating low-temperature precipitation from a model Europan ocean solution of chondritic composition under two freezing regimes. Under ultra-slow freezing, the order of precipitation generally follows that predicted by equilibrium thermodynamics, with the exception that after a significant length of time at low temperature we observe the formation of a highly hydrated sodium magnesium sulfate phase [Na_2_Mg(SO_4_)_2_·16H_2_O], whose identification and structure have only been reported in recent years. Under fast-freezing conditions, on the other hand, all possible phases appear to precipitate at the same time (within the time and temperature resolution of our experiment). With falling temperature they undergo an initial chaotic-like adjustment in their intensities and relative proportions, no doubt due to the initial assemblage being a disequilibrium one. However, below ∼150 K the system appears to settle, with subsequent changes in both the intensities and relative proportions progressing more smoothly. We were able to group the precipitates according to whether their abundances increased, decreased or remained essentially constant. The two main phases were meridianiite (MgSO_4_·11H_2_O) and mirabilite (Na_2_SO_4_·10H_2_O). The Na_2_Mg(SO_4_)_2_·16H_2_O phase was also observed in the fast-freezing experiment and was one of the phases found to increase in abundance with decreasing temperature. On the basis of the increased sinking velocity of dense phases at higher temperatures, we have proposed a mechanism by which the abundance of Na_2_Mg(SO_4_)_2_·16H_2_O could be enhanced at shallow depths within the Europan ice crust.

The precipitation of salts on Europa parallels the precipitation of salts from polar sea ice on Earth, which plays an important ecological role in regulating the habitability of such environments. Since conditions on Europa are widely considered suitable for life, we have discussed the possible biological role that salt precipitates could play on Europa. However, ocean salts on Earth are dominated by sodium chloride, while the salts produced from our model solution are sodium and magnesium sulfates and could lead to significant potential geo-biochemical differences. Although we have concentrated on a single instance of a possible Europan ocean composition, we have discussed how the techniques and methodology of the current work could be applied to further our understanding not only of other Solar System bodies but also of those exoplanets currently being discovered that are likely to host icy oceans.

The identification of materials on planetary surfaces via spectral fitting is limited by the library of plausible candidate materials available with which to do the fitting. As such, laboratory experiments, such the ones described here, are invaluable in providing clues and constraints, since formed phases are determined by nature rather than theory. The observation of Na_2_Mg(SO_4_)_2_·16H_2_O is a prime example. Its existence was discovered rather than predicted and, to the best of our knowledge, it has not previously been suggested as a candidate phase for Europa. Indeed for disequilibrium systems, such as rapid or flash freezing, thermodynamic modelling is likely to be wholly inadequate, leaving laboratory experimentation as the only way forward. Added to this is the surprising complexity of the sulfate and sulfate–chloride systems, particularly at low temperatures where a wide range of hydration states with multiple pathways between them can exist. The discovery of new phases such as Na_2_Mg(SO_4_)_2_·16H_2_O in recent times suggests that there may yet be further discoveries to be made.

## Figures and Tables

**Figure 3:**
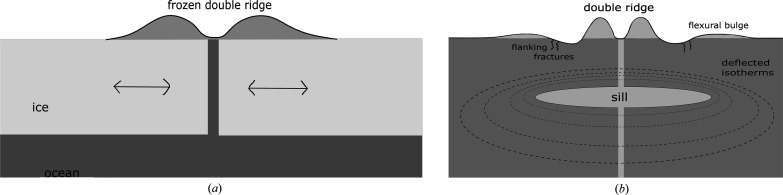
Origin of Europan surface morphologies II: double ridges. A common feature for which many models have been proposed. These include (*a*) tidal squeezing, whereby daily tidal forces cause a crack to open and close, pumping material onto the surface (Greenberg *et al.*, 1998 ▸); and (*b*) cryovolcanism, where a pre-existing crack provides a pathway for fissure eruptions that build the ridges cryoclastically (Kadel *et al.*, 1998 ▸). In terrestrial volcanic systems the amount of subsurface magmatism tends to exceed the amount of surface volcanism. This should also be true on an icy satellite, since the cryomagma (*i.e.* water) is denser than the surface rock (*i.e.* ice). Thus while the ridge is being built as a cryoclastic fissure eruption, the cryomagma should form a sill at a depth corresponding to the neutral buoyancy of water within the ice shell.

**Table 1:** Chemical formula, mineral name, abbreviation and density information (from ICDD PDF-4+ database) of phases identified in the MEOS precipitate

Phase formula	Mineral name	Abbreviation	Density (g cm^−3^)
H_2_O	Water ice	Ih	0.931

Mg phases			
MgSO_4_·5H_2_O	Pentahydrite	MS5	1.929
MgSO_4_·7H_2_O	Epsomite	MS7	1.679
MgSO_4_·11H_2_O	Meridianiite	MS11	1.512

Na phases			
NaCl·2H_2_O	Hydrohalite	NC2	1.654
Na_2_SO_4_·10H_2_O	Mirabilite	NS10	1.465

Ca phases			
CaSO_4_·2H_2_O	Gypsum	CS2	2.307

Na–Mg phases			
Na_2_Mg(SO_4_)_2_·4H_2_O	Blödite	NMS4	2.223
Na_2_Mg(SO_4_)_2_·5H_2_O	Konyaite	NMS5	2.098
Na_12_Mg_7_(SO_4_)_13_·15H_2_O	Loweite	NMS15	2.364
Na_2_Mg(SO_4_)_2_·16H_2_O	–	NMS16	1.623

## Footnotes

1 Prior to 2006, when the correct hydration state was recognized, MgSO_4_·11H_2_O is referred to in the literature as MgSO_4_·12H_2_O.
